# Supplementary material for: The effect of caffeine on tinnitus: Randomized triple-blind placebo-controlled clinical trial
Source: PLoS One. 2021 Sep 20;16(9):e0256275. doi: 10.1371/journal.pone.0256275 (PMC8452027; doi:10.1371/journal.pone.0256275)
Supplement: S1 Appendix — (DOCX) [file pone.0256275.s002.docx]

Appendix 2 - Difference between groups and Correlation with caffeine consumption

**Questionnaire analyses**

| **Difference between groups** | | | | | **Correlation with caffeine consumption** | |
| --- | --- | --- | --- | --- | --- | --- |
| **Questionnaire** | | **Group** | **Mdn (IQR)** | **p-value*** | **Correlation coefficient** | **p-value**** |
| THI phase 1 | | Caffeine | 36.0 (35.0) | 0.784 | -0.041 | 0.719 |
|  |  | Placebo | 32.0 (26.0) |  |  |  |
| THI phase 3 | | Caffeine | 28.0 (35.0) | 0.557 | -0.030 | 0.792 |
|  |  | Placebo | 24.0 (25.0) |  |  |  |
| VAS phase 1 | | Caffeine | 5.0 (4.0) | 0.790 | -0.055 | 0.628 |
|  |  | Placebo | 6.0 (5.0) |  |  |  |
| VAS phase 3 | | Caffeine | 5.0 (4.0) | 0.908 | -0.096 | 0.399 |
|  |  | Placebo | 5.5 (5.0) |  |  |  |
| POMS | TMD phase 1 | Caffeine | -10.0 (31.0) | 0.733 | -0.061 | 0.590 |
|  |  | Placebo | -8.0 (33.5) |  |  |  |
|  | TMD phase 3 | Caffeine | -11.5 (22.0) | 0.450 | -0,071 | 0.530 |
|  |  | Placebo | -11.0 (29.5) |  |  |  |
|  | Tension-anxiety phase 1 | Caffeine | 2.0 (6.5) | 0.640 | 0,018 | 0.871 |
|  |  | Placebo | 1.0 (6.5) |  |  |  |
|  | Tension-anxiety phase 3 | Caffeine | 0.0 (6.0) | 0.965 | -0,054 | 0.634 |
|  |  | Placebo | 0.5 (5.5) |  |  |  |
|  | Depression-despondency phase 1 | Caffeine | 2.0 (10.0) | 0.343 | -0,047 | 0.682 |
|  |  | Placebo | 1.0 (5.0) |  |  |  |
|  | Depression-despondency phase 3 | Caffeine | 1.0 (5.0) | 0.878 | -0.014 | 0.902 |
|  |  | Placebo | 0.5 (3.5) |  |  |  |
|  | Anger-hostility phase 1 | Caffeine | 1.0 (2.0) | 0.815 | -0.061 | 0.588 |
|  |  | Placebo | 0.5 (9.5) |  |  |  |
|  | Anger-hostility phase 3 | Caffeine | 0.5 (2.0) | 0.778 | -0.096 | 0.399 |
|  |  | Placebo | 0.0 (4.0) |  |  |  |
|  | Fatigue–inertia phase 1 | Caffeine | 5.5 (8.5) | 0.880 | -0.069 | 0.543 |
|  |  | Placebo | 4.0 (9.5) |  |  |  |
|  | Fatigue–inertia phase 3 | Caffeine | 4.0 (5.0) | 0.812 | 0.063 | 0.578 |
|  |  | Placebo | 3.0 (7.0) |  |  |  |
|  | Vigor–activity phase 1 | Caffeine | 17.5 (7.0) | 0.443 | -0.013 | 0.908 |
|  |  | Placebo | 18.5 (5.5) |  |  |  |
|  | Vigor–activity phase 3 | Caffeine | 18.5 (5.5) | 0.091 | -0.017 | 0.880 |
|  |  | Placebo | 16.5 (9.5) |  |  |  |
|  | Mental confusion-perplexity phase 1 | Caffeine | 0.0 (4.0) | 0.179 | -0.013 | 0.908 |
|  |  | Placebo | 0.0 (5.5) |  |  |  |
|  | Mental confusion-perplexity phase 3 | Caffeine | 0.0 (3.0) | 0.831 | -0.093 | 0.410 |
|  |  | Placebo | -0.5 (5.0) |  |  |  |

* p-value of the non-parametric Mann-Whitney test** p-value of the spearman correlation test

**Psychoacoustics analysis**

|  |  |  | | **Difference between group differences** | | | **Correlation with caffeine consumption** | |  | **Difference between group differences** | | | **Correlation with caffeine consumption** | |
| --- | --- | --- | --- | --- | --- | --- | --- | --- | --- | --- | --- | --- | --- | --- |
|  | **Group** |  | | **Mdn (IQR)** | **p-value*** | | **CC** | **p-value** |  | **Mdn (IQR)** | **p-value** | | **CC** | **p-value**** |
| **Acufenometry** | | | | | | | | | | | | | | |
| Loudness phase 1 | Caffeine | | | 6.0 (7.5) | | 0.498 | 0.222 | 0.848 |  |  | |  |  |  |
|  | Placebo | | | 5.0 (8.0) | |  |  |  |  |  | |  |  |  |
| Loudness phase 3 | Caffeine | | | 5.0 (5.5) | | 0.376 | 0.005 | 0.967 |  |  | |  |  |  |
|  | Placebo | | | 5.0 (7.5) | |  |  |  |  |  | |  |  |  |
| MML phase 1 | Caffeine | | | 15.5 (14.5) | | 0.777 | -0.002 | 0.986 |  |  | |  |  |  |
|  | Placebo | | | 15.0 (15.0) | |  |  |  |  |  | |  |  |  |
| MML phase 3 | Caffeine | | | 20.0 (15.0) | | 0.134 | 0.082 | 0.489 |  |  | |  |  |  |
|  | Placebo | | | 15.0 (18.0) | |  |  |  |  |  | |  |  |  |
| **Tonal audiometry** | | | | | | | | | | | | | | |
| 0.25kHz Phase 1 | Caffeine | | Right ear | 15.0 (10.0) | 0.817 | | 0.038 | 0.741 | Left ear | 15.0 (10.0) | | 0.428 | 0.136 | 0.229 |
|  | Placebo | |  | 15.0 (10.0) |  |  |  |  |  | 15.0 (10.0) | |  |  |  |
| 0.25kHz Phase 3 | Caffeine | |  | 15.0 (10.0) | 0.407 | | 0.068 | 0.548 |  | 15.0 (10.0) | | 0.124 | 0.138 | 0.221 |
|  | Placebo | |  | 10.0 (5.0) |  |  |  |  |  | 10.0 (5.0) | |  |  |  |
| 0.5kHz Phase 1 | Caffeine | |  | 20.0 (12.5) | 0.302 | | 0.118 | 0.299 |  | 20.0 (7.5) | | 0.075 | 0.227 | 0.043 |
|  | Placebo | |  | 15.0 (15.0) |  |  |  |  |  | 15.0 (10.0) | |  |  |  |
| 0.5kHz Phase 3 | Caffeine | |  | 17.5 (10.0) | 0.284 | | 0.189 | 0.094 |  | 17.5 (10.0) | | 0.019 | 0.188 | 0.096 |
|  | Placebo | |  | 15.0 (15.0) |  |  |  |  |  | 15.0 (10.0) | |  |  |  |
| 1kHz Phase 1 | Caffeine | |  | 15.0 (15.0) | 0.525 | | 0.128 | 0.257 |  | 15.0 (12.5) | | 0.047 | 0.102 | 0.366 |
|  | Placebo | |  | 12.5 (10.0) |  |  |  |  |  | 10.0 (5.0) | |  |  |  |
| 1kHz Phase 3 | Caffeine | |  | 15.0 (17.5) | 0.497 | | 0.137 | 0.227 |  | 15.0 (12.5) | | 0.058 | 0.168 | 0.136 |
|  | Placebo | |  | 12.5 (10.0) |  |  |  |  |  | 10.0 (10.0) | |  |  |  |
| 2kHz Phase 1 | Caffeine | |  | 15.0 (15.0) | 0.323 | | 0.215 | 0.055 |  | 15.0 (17.5) | | 0.098 | 0.166 | 0.142 |
|  | Placebo | |  | 15.0 (20.0) |  |  |  |  |  | 10.0 (12.5) | |  |  |  |
| 2kHz Phase 3 | Caffeine | |  | 15.0 (15.0) | 0.351 | | 0.187 | 0.096 |  | 15.0 (17.5) | | 0.049 | 0.215 | 0.055 |
|  | Placebo | |  | 10.0 (20.0) |  |  |  |  |  | 10.0 (15.0) | |  |  |  |
| 3kHz Phase 1 | Caffeine | |  | 20.0 (22.5) | 0.413 | | 0.144 | 0.203 |  | 20.0 (20.0) | | 0.045 | 0.167 | 0.139 |
|  | Placebo | |  | 15.0 (20.0) |  |  |  |  |  | 15.0 (15.0) | |  |  |  |
| 3kHz Phase 3 | Caffeine | |  | 20.0 (22.5) | 0.433 | | 0.127 | 0.263 |  | 20.0 (25.0) | | 0.028 | 0.154 | 0.172 |
|  | Placebo | |  | 15.0 (25.0) |  |  |  |  |  | 15.0 (10.0) | |  |  |  |
| 4kHz Phase 1 | Caffeine | |  | 22.5 (25.0) | 0.400 | | 0.148 | 0.191 |  | 22.5 (32.5) | | 0.042 | 0.084 | 0.457 |
|  | Placebo | |  | 17.5 (20.0) |  |  |  |  |  | 20.0 (20.0) | |  |  |  |
| 4kHz Phase 3 | Caffeine | |  | 22.5 (32.5) | 0.327 | | 0.184 | 0.103 |  | 20.0 (30.0) | | 0.027 | 0.086 | 0.450 |
|  | Placebo | |  | 20.0 (20.0) |  |  |  |  |  | 17.5 (15.0) | |  |  |  |
| 6kHz Phase 1 | Caffeine | |  | 17.5 (32.5) | 0.859 | | 0.211 | 0.061 |  | 22.5 (27.5) | | 0.523 | 0.053 | 0.639 |
|  | Placebo | |  | 15.0 (25.0) |  |  |  |  |  | 17.5 (20.0) | |  |  |  |
| 6kHz Phase 3 | Caffeine | |  | 15.0 (30.0) | 0.756 | | 0.144 | 0.206 |  | 22.5 (30.0) | | 0.742 | 0.078 | 0.494 |
|  | Placebo | |  | 15.0 (30.0) |  |  |  |  |  | 17.5 (20.0) | |  |  |  |
| 8kHz Phase 1 | Caffeine | |  | 25.0 (40.0) | 0.654 | | 0.217 | 0.055 |  | 27.5 (37.5) | | 0.174 | 0.098 | 0.387 |
|  | Placebo | |  | 25.0 (30.0) |  |  |  |  |  | 22.5 (25.0) | |  |  |  |
| 8kHz Phase 3 | Caffeine | |  | 20.0 (37.5) | 0.829 | | 0.230 | 0.041 |  | 25.0 (35.0) | | 0.205 | 0.107 | 0.344 |
|  | Placebo | |  | 20.0 (30.0) |  |  |  |  |  | 25.0 (27.5) | |  |  |  |
| **High frequency audiometry** | | | | | | | | | | | | | | |
| 9kHz Phase 1 | Caffeine | | Right ear | 22.5 (40.0) | 0.980 | | 0.204 | 0.072 | Left ear | 25.0 (35.0) | | 0.225 | 0.123 | 0.276 |
|  | Placebo | |  | 15.0 (30.0) |  |  |  |  |  | 17.5 (25.0) | |  |  |  |
| 9kHz Phase 3 | Caffeine | |  | 17.5 (35.0) | 0.760 | | 0.182 | 0.109 |  | 25.0 (32.5) | | 0.322 | 0.145 | 0.200 |
|  | Placebo | |  | 15.0 (30.0) |  |  |  |  |  | 20.0 (25.0) | |  |  |  |
| 10kHz Phase 1 | Caffeine | |  | 30.0 (42.5) | 0.572 | | 0.220 | 0.051 |  | 45.0 (40.0) | | 0.501 | 0.158 | 0.165 |
|  | Placebo | |  | 35.0 (40.0) |  |  |  |  |  | 35.0 (30.0) | |  |  |  |
| 10kHz Phase 3 | Caffeine | |  | 30.0 (45.0) | 0.633 | | 0.192 | 0.090 |  | 40.0 (45.0) | | 0.575 | 0.147 | 0.195 |
|  | Placebo | |  | 30.0 (40.0) |  |  |  |  |  | 30.0 (25.0) | |  |  |  |
| 11.2kHz Phase 1 | Caffeine | |  | 35.0 (52.5) | 0.973 | | 0.213 | 0.060 |  | 50.0 (50.0) | | 0.687 | 0.167 | 0.142 |
|  | Placebo | |  | 40.0 (45.0) |  |  |  |  |  | 40.0 (35.0) | |  |  |  |
| 11.2kHz Phase 3 | Caffeine | |  | 37.5 (50.0) | 0.910 | | 0.219 | 0.052 |  | 50.0 (40.0) | | 0.676 | 0.146 | 0.198 |
|  | Placebo | |  | 40.0 (45.0) |  |  |  |  |  | 40.0 (35.0) | |  |  |  |
| 12.5kHz Phase 1 | Caffeine | |  | 40.0 (55.0) | 0.713 | | 0.185 | 0.103 |  | 45.0 (40.0) | | 0.582 | 0.173 | 0.128 |
|  | Placebo | |  | 40.0 (55.0) |  |  |  |  |  | 42.5 (35.0) | |  |  |  |
| 12.5kHz Phase 3 | Caffeine | |  | 45.0 (50.0) | 0.565 | | 0.190 | 0.094 |  | 45.0 (40.0) | | 0.452 | 0.134 | 0.238 |
|  | Placebo | |  | 40.0 (50.0) |  |  |  |  |  | 45.0 (32.5) | |  |  |  |
| 14kHz Phase 1 | Caffeine | |  | 50.0 (45.0) | 0.921 | | 0.217 | 0.060 |  | 45.0 (45.0) | | 0.823 | 0.237 | 0.039 |
|  | Placebo | |  | 47.5 (45.0) |  |  |  |  |  | 50.0 (35.0) | |  |  |  |
| 14kHz Phase 3 | Caffeine | |  | 50.0 (45.0) | 0.851 | | 0.232 | 0.044 |  | 45.0 (50.0) | | 0.807 | 0.230 | 0.046 |
|  | Placebo | |  | 50.0 (40.0) |  |  |  |  |  | 40.0 (35.0) | |  |  |  |
| 16kHz Phase 1 | Caffeine | |  | 55.0 (35.0) | 0.898 | | 0.175 | 0.134 |  | 55.0 (30.0) | | 0.532 | 0.201 | 0.086 |
|  | Placebo | |  | 55.0 (30.0) |  |  |  |  |  | 55.0 (40.0) | |  |  |  |
| 16kHz Phase 3 | Caffeine | |  | 55.0 (35.0) | 0.602 | | 0.210 | 0.071 |  | 50.0 (25.0) | | 0.798 | 0.234 | 0.045 |
|  | Placebo | |  | 50.0 (25.0) |  |  |  |  |  | 55.0 (35.0) | |  |  |  |
| 18kHz Phase 1 | Caffeine | |  | 25.0 (20.0) | 0.228 | | 0.194 | 0.121 |  | 25.0 (20.0) | | 0.901 | 0.189 | 0.128 |
|  | Placebo | |  | 30.0 (10.0) |  |  |  |  |  | 25.0 (15.0) | |  |  |  |
| 18kHz Phase 3 | Caffeine | |  | 25.0 (10.0) | 0.630 | | 0.220 | 0.079 |  | 25.0 (20.0) | | 0.642 | 0.281 | 0.022 |
|  | Placebo | |  | 25.0 (10.0) |  |  |  |  |  | 25.0 (15.0) | |  |  |  |

* p-value of the non-parametric Mann-Whitney test** p-value of the spearman correlation test

**Electroacoustic analysis**

|  |  | | |  | | | **Difference between group differences** | | | | **Correlation with caffeine consumption** | | |  | | **Difference between group differences** | | | | **Correlation with caffeine consumption** | | | | |
| --- | --- | --- | --- | --- | --- | --- | --- | --- | --- | --- | --- | --- | --- | --- | --- | --- | --- | --- | --- | --- | --- | --- | --- | --- |
|  | **Group** | | |  | | | **Mdn (IQR)** | | **p-value*** | | **CC** | **p-value**** | |  | | **Mdn (IQR)** | | **p-value*** | | **CC** | | **p-value**** | | |
| **Distortion product otoacoustic emission (DPOAE) – Right ear** | | | | | | | | | | | | | | | | | | | | | | | | |
| 1.5kHz Phase 1 | | Caffeine | | | S/N | | 10.5 (11.0) | | 0.444 | | 0.021 | | 0.852 | | S | | 0.0 (10.5) | 0.208 | | -0.051 | | | | 0.650 |
|  |  | Placebo | | |  |  | 8.5 (12.0) | |  |  |  |  |  |  |  |  | -1.5 (14.0) |  |  |  |  |  |  |  |
| 1.5kHz Phase 3 | | Caffeine | | |  |  | 10.5 (10.5) | | 0.401 | | -0.069 | | 0.542 | |  |  | -1.5 (12.5) | 0.527 | | -0.108 | | | | 0.340 |
|  |  | Placebo | | |  |  | 9.0 (14.5) | |  |  |  |  |  |  |  |  | -3.5 (15.5) |  |  |  |  |  |  |  |
| 2kHz Phase 1 | | Caffeine | | |  |  | 12.0 (14.5) | | 0.912 | | -0.092 | | 0.419 | |  |  | 1.0 (12.0) | 0.736 | | -0.162 | | | | 0.151 |
|  |  | Placebo | | |  |  | 12.0 (14.5) | |  |  |  |  |  |  |  |  | 1.0 (13.0) |  |  |  |  |  |  |  |
| 2kHz Phase 3 | | Caffeine | | |  |  | 11.0 (15.5) | | 0.942 | | -0.179 | | 0.112 | |  |  | -2.0 (15.5) | 0.946 | | -0.190 | | | | 0.091 |
|  |  | Placebo | | |  |  | 14.0 (17.5) | |  |  |  |  |  |  |  |  | 0.0 (19.0) |  |  |  |  |  |  |  |
| 3kHz Phase 1 | | Caffeine | | |  |  | 13.5 (15.5) | | 0.950 | | -0.053 | | 0.638 | |  |  | -4.5 (16.0) | 0.965 | | -0.098 | | | | 0.386 |
|  |  | Placebo | | |  |  | 13.0 (13.5) | |  |  |  |  |  |  |  |  | -2.5 (11.0) |  |  |  |  |  |  |  |
| 3kHz Phase 3 | | Caffeine | | |  |  | 12.5 (18.5) | | 0.316 | | -0.008 | | 0.946 | |  |  | -5.5 (18.5) | 0.321 | | -0.033 | | | | 0.771 |
|  |  | Placebo | | |  |  | 15.5 (14.0) | |  |  |  |  |  |  |  |  | -2.5 (15.5) |  |  |  |  |  |  |  |
| 4kHz Phase 1 | | Caffeine | | |  |  | 8.0 (15.5) | | 0.563 | | -0.142 | | 0.208 | |  |  | -11.0 (17.0) | 0.557 | | -0.172 | | | | 0.128 |
|  |  | Placebo | | |  |  | 10.5 (16.5) | |  |  |  |  |  |  |  |  | -9.5 (13.5) |  |  |  |  |  |  |  |
| 4kHz Phase 3 | | Caffeine | | |  |  | 8.0 (15.5) | | 0.590 | | -0.0159 | | 0.160 | |  |  | -12.5 (15.5) | 0.582 | | -0.163 | | | | 0.148 |
|  |  | Placebo | | |  |  | 9.0 (11.0) | |  |  |  |  |  |  |  |  | -11.0 (12.5) |  |  |  |  |  |  |  |
| 5kHz Phase 1 | | Caffeine | | |  |  | 5.0 (12.0) | | 0.820 | | -0.134 | | 0.237 | |  |  | -16.0 (12.0) | 0.737 | | -0.111 | | | | 0.328 |
|  |  | Placebo | | |  |  | 3.0 (13.0) | |  |  |  |  |  |  |  |  | -17.0 (13.0) |  |  |  |  |  |  |  |
| 5kHz Phase 3 | | Caffeine | | |  |  | 5.0 (12.0) | | 0.980 | | -0.109 | | 0.335 | |  |  | -15.0 (12.0) | 0.893 | | -0.097 | | | | 0.392 |
|  |  | Placebo | | |  |  | 5.5 (11.0) | |  |  |  |  |  |  |  |  | -14.0 (11.0) |  |  |  |  |  |  |  |
| 6kHz Phase 1 | | Caffeine | | |  |  | 0.0 (10.5) | | 0.927 | | -0.157 | | 0.164 | |  |  | -20.0 (12.5) | 0.851 | | -0.138 | | | | 0.221 |
|  |  | Placebo | | |  |  | 0.0 (11.5) | |  |  |  |  |  |  |  |  | -20.0 (11.5) |  |  |  |  |  |  |  |
| 6kHz Phase 3 | | Caffeine | | |  |  | 0.0 (12.0) | | 0.714 | | -0.101 | | 0.373 | |  |  | -20.0 (12.0) | 0.955 | | -0.091 | | | | 0.422 |
|  |  | Placebo | | |  |  | 0.5 (12.0) | |  |  |  |  |  |  |  |  | -19.5 (12.0) |  |  |  |  |  |  |  |
| **Distortion product otoacoustic emission (DPOAE) – Left ear** | | | | | | | | | | | | | | | | | | | | | | | | |
| 1.5kHz Phase 1 | | | Caffeine | | | S/N | | 9.0 (13.5) | | 0.897 | -0.109 | | 0.335 | S | | | -1.0 (9.5) | | 0.230 | | -0.117 | | 0.302 | |
|  |  |  | Placebo | | |  |  | 9.0 (11.5) | |  |  |  |  |  |  |  | -2.0 (12.5) | |  |  |  |  |  |  |
| 1.5kHz Phase 3 | | | Caffeine | | |  |  | 9.0 (8.0) | | 0.696 | 0.001 | | 0.990 |  |  |  | -0.5 (11.5) | | 0.458 | | -0.064 | | 0.575 | |
|  |  |  | Placebo | | |  |  | 9.0 (9.0) | |  |  |  |  |  |  |  | -1.5 (12.0) | |  |  |  |  |  |  |
| 2kHz Phase 1 | | | Caffeine | | |  |  | 12.5 (15.5) | | 0.429 | -0.166 | | 0.141 |  |  |  | 1.0 (14.5) | | 0.958 | | -0.151 | | 0.182 | |
|  |  |  | Placebo | | |  |  | 13.0 (9.5) | |  |  |  |  |  |  |  | 1.5 (9.0) | |  |  |  |  |  |  |
| 2kHz Phase 3 | | | Caffeine | | |  |  | 13.5 (12.5) | | 0.380 | -0.051 | | 0.651 |  |  |  | 1.0 (14.5) | | 0.977 | | -0.090 | | 0.428 | |
|  |  |  | Placebo | | |  |  | 15.5 (9.0) | |  |  |  |  |  |  |  | 0.5 (11.0) | |  |  |  |  |  |  |
| 3kHz Phase 1 | | | Caffeine | | |  |  | 8.5 (18.0) | | 0.047 | -0.045 | | 0.691 |  |  |  | -5.0 (16.5) | | 0.136 | | -0.098 | | 0.389 | |
|  |  |  | Placebo | | |  |  | 16.5 (12.0) | |  |  |  |  |  |  |  | -1.5 (13.0) | |  |  |  |  |  |  |
| 3kHz Phase 3 | | | Caffeine | | |  |  | 12.5 (16.0) | | 0.043 | 0.000 | | 0.998 |  |  |  | -5.5 (12.5) | | 0.080 | | -0.027 | | 0.810 | |
|  |  |  | Placebo | | |  |  | 17.0 (9.5) | |  |  |  |  |  |  |  | -2.0 (10.5) | |  |  |  |  |  |  |
| 4kHz Phase 1 | | | Caffeine | | |  |  | 7.0 (15.5) | | 0.069 | -0.084 | | 0.461 |  |  |  | -11.5 (15.5) | | 0.125 | | -0.111 | | 0.326 | |
|  |  |  | Placebo | | |  |  | 11.5 (11.0) | |  |  |  |  |  |  |  | -8.5 (11.0) | |  |  |  |  |  |  |
| 4kHz Phase 3 | | | Caffeine | | |  |  | 6.5 (13.5) | | 0.181 | -0.037 | | 0.747 |  |  |  | -13.5 (13.5) | | 0.162 | | -0.055 | | 0.627 | |
|  |  |  | Placebo | | |  |  | 11.0 (13.0) | |  |  |  |  |  |  |  | -9.0 (13.5) | |  |  |  |  |  |  |
| 5kHz Phase 1 | | | Caffeine | | |  |  | 4.0 (11.5) | | 0.435 | -0.102 | | 0.368 |  |  |  | -15.5 (12.0) | | 0.644 | | -0.146 | | 0.196 | |
|  |  |  | Placebo | | |  |  | 6.0 (10.5) | |  |  |  |  |  |  |  | -14.0 (10.5) | |  |  |  |  |  |  |
| 5kHz Phase 3 | | | Caffeine | | |  |  | 1.0 (12.0) | | 0.658 | -0.22 | | 0.843 |  |  |  | -19.5 (12.0) | | 0.637 | | -0.043 | | 0.705 | |
|  |  |  | Placebo | | |  |  | 6.5 (12.0) | |  |  |  |  |  |  |  | -13.5 (12.5) | |  |  |  |  |  |  |
| 6kHz Phase 1 | | | Caffeine | | |  |  | 0.0 (6.5) | | 0.580 | -0.043 | | 0.708 |  |  |  | -20.0 (6.5) | | 0.690 | | -0.066 | | 0.559 | |
|  |  |  | Placebo | | |  |  | 0.0 (9.5) | |  |  |  |  |  |  |  | -20.0 (9.5) | |  |  |  |  |  |  |
| 6kHz Phase 3 | | | Caffeine | | |  |  | 0.0 (7.5) | | 0.855 | -0.023 | | 0.842 |  |  |  | -20.0 (7.5) | | 0.892 | | -0.036 | | 0.753 | |
|  |  |  | Placebo | | |  |  | 0.0 (7.5) | |  |  |  |  |  |  |  | -20.0 (8.0) | |  |  |  |  |  |  |
| **Transient otoacoustic emission (TOAE) – Right ear** | | | | | | | | | | | | | | | | | | | | | | | | |
| 1.5kHz Phase 1 | | | Caffeine | | | S/N | | 8.0 (8.0) | | 0.223 | -0.017 | | 0.884 | S | | | -3.5 (10.0) | | 0.874 | | -0.069 | | 0.541 | |
|  |  |  | Placebo | | |  |  | 9.0 (7.0) | |  |  |  |  |  |  |  | -4.5 (15.0) | |  |  |  |  |  |  |
| 1.5kHz Phase 3 | | | Caffeine | | |  |  | 7.0 (7.5) | | 0.278 | -0.056 | | 0.624 |  |  |  | -6.0 (11.0) | | 0.900 | | -106 | | 0.351 | |
|  |  |  | Placebo | | |  |  | 8.5 (7.0) | |  |  |  |  |  |  |  | -4.0 (14.5) | |  |  |  |  |  |  |
| 2kHz Phase 1 | | | Caffeine | | |  |  | 7.0 (7.5) | | 0.889 | -0.064 | | 0.573 |  |  |  | -7.5 (8.5) | | 0.661 | | -0.097 | | 0.392 | |
|  |  |  | Placebo | | |  |  | 6.5 (6.5) | |  |  |  |  |  |  |  | -8.0 (14.5) | |  |  |  |  |  |  |
| 2kHz Phase 3 | | | Caffeine | | |  |  | 6.0 (7.0) | | 0.900 | -0.121 | | 0.286 |  |  |  | -8.5 (9.5) | | 0.810 | | -0.095 | | 0.401 | |
|  |  |  | Placebo | | |  |  | 6.5 (6.5) | |  |  |  |  |  |  |  | -9.0 (13.5) | |  |  |  |  |  |  |
| 2.5kHz Phase 1 | | | Caffeine | | |  |  | 6.0 (8.0) | | 0.606 | -0.162 | | 0.151 |  |  |  | -13.0 (10.5) | | 0.780 | | -0.079 | | 0.488 | |
|  |  |  | Placebo | | |  |  | 6.0 (7.5) | |  |  |  |  |  |  |  | -12.0 (11.5) | |  |  |  |  |  |  |
| 2.5kHz Phase 3 | | | Caffeine | | |  |  | 5.5 (9.0) | | 0.985 | -0.115 | | 0.309 |  |  |  | -14.0 (9.5) | | 0.557 | | -0.077 | | 0.498 | |
|  |  |  | Placebo | | |  |  | 6.5 (7.0) | |  |  |  |  |  |  |  | -14.0 (13.0) | |  |  |  |  |  |  |
| 3kHz Phase 1 | | | Caffeine | | |  |  | 5.5 (8.5) | | 0.862 | -0.126 | | 0.265 |  |  |  | -17.5 (9.5) | | 0.550 | | -0.092 | | 0.430 | |
|  |  |  | Placebo | | |  |  | 5.0 (7.5) | |  |  |  |  |  |  |  | -17.0 (11.5) | |  |  |  |  |  |  |
| 3kHz Phase 3 | | | Caffeine | | |  |  | 6.0 (8.5) | | 0.765 | -0.130 | | 0.249 |  |  |  | -16.0 (13.5) | | 0.418 | | -0.129 | | 0.253 | |
|  |  |  | Placebo | | |  |  | 6.0 (7.0) | |  |  |  |  |  |  |  | -16.0 (11.0) | |  |  |  |  |  |  |
| 3.5kHz Phase 1 | | | Caffeine | | |  |  | 2.5 (6.0) | | 0.732 | -0.066 | | 0.563 |  |  |  | -17.0 (8.0) | | 0.881 | | -0.072 | | 0.528 | |
|  |  |  | Placebo | | |  |  | 2.0 (7.0) | |  |  |  |  |  |  |  | -17.5 (12.5) | |  |  |  |  |  |  |
| 3.5kHz Phase 3 | | | Caffeine | | |  |  | 3.0 (6.0) | | 0.639 | -0.139 | | 0.217 |  |  |  | -18.5 (9.0) | | 0.700 | | -0.0145 | | 0.199 | |
|  |  |  | Placebo | | |  |  | 3.0 (8.0) | |  |  |  |  |  |  |  | -17.0 (12.0) | |  |  |  |  |  |  |
| 4kHz Phase 1 | | | Caffeine | | |  |  | -1.0 (2.5) | | 0.057 | -0.083 | | 0.462 |  |  |  | -18.0 (5.5) | | 0.300 | | -0.154 | | 0.172 | |
|  |  |  | Placebo | | |  |  | 1.0 (5.5) | |  |  |  |  |  |  |  | -18.0 (10.5) | |  |  |  |  |  |  |
| 4kHz Phase 3 | | | Caffeine | | |  |  | -1.0 (6.5) | | 0.159 | -0.079 | | 0.486 |  |  |  | -20.0 (8.5) | | 0.560 | | -0.171 | | 0.129 | |
|  |  |  | Placebo | | |  |  | -1.0 (6.0) | |  |  |  |  |  |  |  | -18.0 (10.0) | |  |  |  |  |  |  |
| **Transient otoacoustic emission (TOAE) – Left ear** | | | | | | | | | | | | | | | | | | | | | | | | |
| 1.5kHz Phase 1 | | | Caffeine | | | S/N | | 6.0 (7.0) | | 0.609 | -0.102 | | 0.367 | S | | | -6.0 (12.5) | | 0.942 | | 0.019 | | 0.866 | |
|  |  |  | Placebo | | |  |  | 6.5 (6.0) | |  |  |  |  |  |  |  | -5.0 (12.0) | |  |  |  |  |  |  |
| 1.5kHz Phase 3 | | | Caffeine | | |  |  | 7.0 (10.0) | | 0.824 | -0.166 | | 0.142 |  |  |  | -6.0 (12.5) | | 0.391 | | -0.084 | | 0.457 | |
|  |  |  | Placebo | | |  |  | 8.8 (5.5) | |  |  |  |  |  |  |  | -4.5 (12.0) | |  |  |  |  |  |  |
| 2kHz Phase 1 | | | Caffeine | | |  |  | 5.5 (7.0) | | 0.122 | -0.006 | | 0.958 |  |  |  | -10.0 (11.0) | | 0.843 | | 0.125 | | 0.271 | |
|  |  |  | Placebo | | |  |  | 7.0 (3.0) | |  |  |  |  |  |  |  | -7.0 (11.5) | |  |  |  |  |  |  |
| 2kHz Phase 3 | | | Caffeine | | |  |  | 6.5 (8.0) | | 0.272 | 0.039 | | 0.730 |  |  |  | -9.5 (11.5) | | 0.380 | | 0.064 | | 0.575 | |
|  |  |  | Placebo | | |  |  | 8.0 (6.0) | |  |  |  |  |  |  |  | -7.0 (9.0) | |  |  |  |  |  |  |
| 2.5kHz Phase 1 | | | Caffeine | | |  |  | 5.0 (6.0) | | 0.132 | -0.029 | | 0.798 |  |  |  | -13.5 (9.0) | | 0.586 | | 0.076 | | 0.503 | |
|  |  |  | Placebo | | |  |  | 7.0 (7.0) | |  |  |  |  |  |  |  | -12.5 (9.5) | |  |  |  |  |  |  |
| 2.5kHz Phase 3 | | | Caffeine | | |  |  | 5.0 (6.5) | | 0.096 | 0.038 | | 0.741 |  |  |  | -15.5 (10.5) | | 0.126 | | -0.019 | | 0.868 | |
|  |  |  | Placebo | | |  |  | 7.0 (6.5) | |  |  |  |  |  |  |  | -11.5 (8.0) | |  |  |  |  |  |  |
| 3kHz Phase 1 | | | Caffeine | | |  |  | 4.0 (9.0) | | 0.398 | -0.045 | | 0.689 |  |  |  | -18.0 (12.5) | | 0.912 | | 0.022 | | 0.846 | |
|  |  |  | Placebo | | |  |  | 5.5 (5.5) | |  |  |  |  |  |  |  | -16.5 (12.0) | |  |  |  |  |  |  |
| 3kHz Phase 3 | | | Caffeine | | |  |  | 4.5 (8.5) | | 0.278 | 0.025 | | 0.827 |  |  |  | -18.0 (13.5) | | 0.586 | | -0.051 | | 0.652 | |
|  |  |  | Placebo | | |  |  | 5.5 (7.5) | |  |  |  |  |  |  |  | -17.0 (8.5) | |  |  |  |  |  |  |
| 3.5kHz Phase 1 | | | Caffeine | | |  |  | 1.0 (4.0) | | 0.030 | -0.043 | | 0.707 |  |  |  | -19.0 (11.0) | | 0.251 | | 0.018 | | 0.874 | |
|  |  |  | Placebo | | |  |  | 5.0 (8.5) | |  |  |  |  |  |  |  | -16.0 (14.0) | |  |  |  |  |  |  |
| 3.5kHz Phase 3 | | | Caffeine | | |  |  | 2.0 (5.5) | | 0.024 | 0.007 | | 0.949 |  |  |  | -18.5 (11.5) | | 0.192 | | -0.038 | | 0.735 | |
|  |  |  | Placebo | | |  |  | 5.0 (7.5) | |  |  |  |  |  |  |  | -17.5 (13.0) | |  |  |  |  |  |  |
| 4kHz Phase 1 | | | Caffeine | | |  |  | -1.0 (6.5) | | 0.013 | 0.127 | | 0.263 |  |  |  | -20.0 (6.5) | | 0.091 | | 0.053 | | 0.638 | |
|  |  |  | Placebo | | |  |  | 1.5 (6.5) | |  |  |  |  |  |  |  | -16.0 (8.5) | |  |  |  |  |  |  |
| 4kHz Phase 3 | | | Caffeine | | |  |  | 0.0 (5.0) | | 0.451 | 0.104 | | 0.360 |  |  |  | -18.5 (9.0) | | 0.257 | | -0.047 | | 0.682 | |
|  |  |  | Placebo | | |  | | 1.0 (5.0) | |  |  |  |  |  | | | -16.5 (9.5) | |  |  |  |  |  |  |

* p-value of the non-parametric Mann-Whitney test ** p-value of the spearman correlation test
