# Supplementary material for: The effect of caffeine on tinnitus: Randomized triple-blind placebo-controlled clinical trial
Source: PLoS One. 2021 Sep 20;16(9):e0256275. doi: 10.1371/journal.pone.0256275 (PMC8452027; doi:10.1371/journal.pone.0256275)
Supplement: S2 Appendix — (PDF) [file pone.0256275.s003.pdf]

PARECER CONSUBSTANCIADO DO CEP

DADOS DO PROJETO DE PESQUISA

Título da Pesquisa: O efeito da cafeína na autopercepção do zumbido
Pesquisador: Alleluia Lima Losno Ledesma
Área Temática:
Versão: 2
CAAE: 63290116.2.0000.0030
Instituição Proponente: Faculdade de Ciências da Saúde da Universidade de Brasília
Patrocinador Principal: Financiamento Próprio

DADOS DO PARECER

Número do Parecer: 2.031.285

Apresentação do Projeto:
“Resumo:
Introdução: Alguns autores sugerem que, pelo fato de a cafeína ser um estimulante do sistema nervoso central, seu uso pode provocar uma alteração na auto percepção do zumbido. Ao mesmo tempo a experiência clínica sugere que uma dieta que restringe o uso de cafeína não modifica o incômodo do paciente em relação ao zumbido, podendo levar a um aumento do desconforto e a uma colaboração diminuída do paciente às recomendações médicas. Enquanto não se chega a um consenso cada serviço adota as recomendações que lhe parecem convenientes, uma vez que na literatura o tema ainda é controverso, não trazendo, portanto, definições acerca da influência desta substância sobre a queixa de zumbido. Objetivos: Analisar a influência da cafeína na auto percepção do zumbido. Métodos: Serão selecionados 80 indivíduos jovens e saudáveis, sem queixas outras auditivas ou vestibulares, exceto o zumbido. Os sujeitos serão divididos aleatoriamente em dois grupos: cafeína e placebo. Será orientada uma dieta que restrinja a cafeína por 24 horas e os indivíduos serão submetidos a exames e questionários em dois momentos: basal e após a ingestão de cápsulas. ”

“Introdução:
O zumbido é um sintoma definido como a percepção auditiva na ausência de uma fonte sonora

externa (Coelho et al., 2004; Almeida et al., 2009).Segundo a Organização Mundial da Saúde (OMS), 278 milhões de pessoas têm zumbido, aproximadamente 15% da população mundial. Esta prevalência aumenta para 33% entre os indivíduos com mais de 60 anos de idade (Jastebroff, 1980; Coelho et al., 2004; Pinto et al., 2010). No Brasil, estima-se que mais de 28 milhões de indivíduos sejam portadores de zumbido, tornando-o um problema de saúde pública (Sanchez et al., 2002). A cafeína (1, 3, 7-trimetilxantina) é um estimulante do sistema nervoso central pertencente ao grupo das metilxantinas (CAMARGO e TOLEDO, 1998; MYCEK, HARVEY e CHAMPE, 1998; SILVA, 1998; KATZUNG, 2005; ALVES, CASAL e OLIVEIRA, 2009). O seu metabolismo vem sendo estudado desde 1850, antes mesmo da sua caracterização química (1895), e ainda hoje não se têm conclusões definitivas (CAMARGO, 1996). Devido à cafeína ser um estimulante central, alguns autores sugerem que seu uso pode provocar uma alteração na auto percepção do zumbido. Ao mesmo tempo a experiência clínica sugere que uma dieta que restringe o uso de cafeína não modifica o incômodo do paciente em relação ao zumbido, podendo levar a um aumento do desconforto e a uma colaboração diminuída do paciente às recomendações médicas. Enquanto não se chega a um consenso cada serviço adota as recomendações que lhe parecem convenientes, uma vez que na literatura o tema ainda é controverso, não trazendo, portanto, definições acerca da influência desta substância sobre a queixa de zumbido.”

“Hipótese: A cafeína não influencia na autopercepção do zumbido”

“Metodologia Proposta:
Os participantes responderão a um questionário on line buscando identificar aqueles que se enquadram nos critérios de seleção do estudo e o Questionário de Hábitos Alimentares (adaptado de Camargo 1996) buscando conhecer a quantidade de cafeína ingerida diariamente pelos participantes. A partir das informações obtidas no Questionário de Hábitos Alimentares será calculada a quantidade de cafeína ingerida diariamente pelos participantes, utilizando os valores encontrados no estudo de Camargo (1996) quanto ao volume dos recipientes e ao teor de cafeína de cada produto. Os participantes serão, então, classificados de acordo com a quantidade de cafeína que costumam ingerir diariamente, como proposto por Schreiber et al. (1988): consumo esporádico (menos de 100mg), leve (de 100 a 299mg), moderado (300 a 499mg). Os que tiverem consumo maior ou igual a 500mg/ dia (intenso ou muito intenso) não participarão das demais etapas do estudo. Aqueles participantes que não se enquadrarem nos critérios de seleção terão suas dúvidas esclarecidas e lhes será explicado os motivos da exclusão. Quando os motivos da

exclusão envolverem queixas auditivas e/ou vestibulares os participantes serão orientados a procurar um médico otorrinolaringologista para uma avaliação detalhada. Os que se enquadrarem nos critérios de seleção serão orientados a se abster de alimentos/ produtos que contenham cafeína 24 horas antes da participação no estudo. Ao chegar à clínica os participantes serão questionados sobre a obediência às recomendações para os exames e aqueles que declararem não ter seguido, porém, ainda desejarem participar do estudo, terão novo encontro agendado. Os que tiverem seguido as recomendações responderão ao POMS, ao THI e à EVA, na ordem descrita, em seguida será realizada audiometria tonal, acufenometria e Emissões Otoacústicas Produto de Distorção. Após este primeiro momento, os participantes receberam cápsulas que pode conter placebo ou cafeína (300mg) com pequena quantidade de água. Uma hora após a ingestão da mesma responderão novamente ao POMS, ao THI e à EVA e serão submetidos novamente à acufenometria e às Emissões Otoacústicas Produto de Distorção. Um colaborador do estudo controlará os participantes que receberão cafeína e os que receberão placebo, não sendo informado aos participantes, pesquisador e estatístico quais participantes pertencem a cada grupo."

Critério de Inclusão:
"Participarão do estudo voluntários que sejam maiores de 18 anos e possuam queixa de zumbido."
Critério de Exclusão:
"Serão excluídos do estudo os sujeitos que alterações metabólicas, alterações hormonais, distúrbios psíquicos, doenças neurológicas, que utilizem medicamentos de uso contínuo, fumantes, alcoolistas e usuários de drogas ilícitas. "

"Metodologia de Análise de Dados:
Medidas no basal, nos dois grupos, serão comparadas usando o teste de Qui-quadrado ou teste exato de Fisher (no caso de frequências esperadas menores do que 1) para variáveis qualitativas. No caso de variáveis quantitativas com distribuição gaussiana, em ambos os grupos, será empregado o teste t de Student, ou o teste não paramétrico de Mann-Whitney, para aquelas sem distribuição gaussiana. Valores pós-intervenção das variáveis (acufenometria) serão comparados entre grupos empregando-se um modelo de análise de covariância (ANCOVA). Estes valores serão considerados, no modelo estatístico, como variável dependente, o tipo de intervenção será a

variável independente e os valores no basal das variáveis serão considerados como covariável. Considerar-se-á significativo p < 0,05. A análise será realizada empregando-se o aplicativo SAS 9.4 (SAS Institute, Inc., 1999)."

"Desfecho Primário:
Serão comparados os escores os resultados da acufenometria antes e após o uso da cafeína/placebo visando identificar a impressão geral do incômodo relacionado ao zumbido nos participantes nos dois momentos.
Desfecho Secundário:
Analisar os escores antes e após o uso da cafeína/placebo em cada um dos exames e questionários separadamente, observando quais os aspectos que sofreram ou não influência da substância
"Tamanho da Amostra no Brasil: 80"

Objetivo da Pesquisa:
"Objetivo Primário:
Analisar a influência da cafeína na auto percepção do zumbido.
Objetivo Secundário:
Analisar a influência da cafeína na Acufenometria
Analisar a influência da cafeína no Tinnitus Handicap Inventory (THI)
Analisar a influência da cafeína na Escala Visual Analógica (EVA)
Analisar a influência da cafeína nas Emissões Otoacústicas Produto de Distorção (EOA-PD)
Analisar as alterações de humor antes da realização do exame."

Avaliação dos Riscos e Benefícios:
"Riscos:
Os riscos inerentes ao estudo são os comuns na abstinência da cafeína: cefaléia, fadiga, letargia, "sintomas semelhantes ao da gripe" e distúrbios do humor. Esses sintomas são passageiros e facilmente controláveis através de analgésicos. Além disso, o indivíduo deverá dispor de tempo para realização dos testes, que é cerca de 3 horas no total.
Benefícios:
Como benefícios, o indivíduo terá a oportunidade de ter uma avaliação do sistema auditivo, além de ser orientado quanto ao funcionamento do mesmo, bem como de ser atendido por um

especialista caso haja alteração no exame convencional (com restrição da cafeína).”

Comentários e Considerações sobre a Pesquisa:

Trata-se de projeto da pesquisadora Alleluia Lima Losno Ledesma, do Programa de Pós-Gradução em Ciências da Saúde da Faculdade de Ciências da Saúde da UnB, sob a orientação do Dr. Fayez Bahmad Junior.

Local de realização do projeto: Instituto Brasiliense de Otorrinolaringologia.

Patrocinador principal: Financiamento próprio, com a utilização de equipamentos do local de realização do projeto.

Considerações sobre os Termos de apresentação obrigatória:

PB_INFORMAÇÕES_BÁSICAS_DO_PROJETO_804358.pdf – anexado em 05 abr 2017 – projeto com informações básicas, com modificações no texto a fim de atender as pendencias apontadas em análise de versão anterior, bem como as modificações no Cronograma de Execução e Orçamento Financeiro.

cartaResposta.docx – anexado em 05 abr 2017 – carta resposta ao CEP/FS, sem data e sem assinatura, com a descrição das modificações que foram executadas, ou a complementação de informações, a fim de atender as pendencias apontadas em análise de versão anterior.

ProjetoCEP2.docx – anexado em 05 abr 2017 – projeto completo apresentado com as modificações e complementações necessárias para o atendimento às pendencias verificadas em análise de versão anterior.

TCLE2.docx – anexado em 05 abr 2017 – documento modificado em atendimento às pendencias verificadas em análise de versão anterior.

Recomendações:

Não se aplica.

Conclusões ou Pendências e Lista de Inadequações:

Foram verificadas as pendências apontadas pelo Parecer Consubstanciado nº
1.962.542, de 13 de março de 2017:

1 – os documentos "ProjetoCEP.docx" e o "PB_INFORMAÇÕES_BÁSICAS_DO_ PROJETO_804358.pdf"

devem conter as informações (Res. CNS 466/2012, itens II.3; II.10; II.22; III.1, d; III.2 a, b; IV.1 b; IV.3 e):
1.a) riscos associados à ingestão da cafeína, bem como os procedimentos para evitá-los, minimizá-los e providencias caso ocorram, bem como a justificativa da dosagem a ser oferecida ao participante da pesquisa;

RESPOSTA: "1.a) Riscos associados à ingestão da cafeína, bem como os procedimentos para evitá-los, minimizá-los e providencias caso ocorram, bem como a justificativa da dosagem a ser oferecida ao participante da pesquisa; Página 20, Ítem 4.6 Análise dos riscos e benefícios "A ingestão da cafeína (300mg) pode gerar aumento do estado de vigília e sensação de alerta, que tem remissão espontânea após o tempo de ação da substância que varia de 2 a 4 horas. Essa concentração refere-se a um consumo moderado de cafeína, sendo o consumo médio habitual nos brasileiros. "
ANÁLISE: PENDÊNCIA ATENDIDA.

1.b) descrição do placebo a ser utilizado e dosagem, os riscos associados à sua ingestão, bem como os procedimentos para evitá-los, minimizá-los e providencias caso ocorram;

RESPOSTA: "1.b) Descrição do placebo a ser utilizado e dosagem, os riscos associados à sua ingestão, bem como os procedimentos para evitá-los, minimizá-los e providencias caso ocorram Página 20, Ítem 4.6 Análise dos riscos e benefícios "O placebo utilizado será o amido de milho (1g), sendo que a sua ingestão não gera nenhum desconforto em pessoas que não apresentem alergia à substância.""
ANÁLISE: PENDÊNCIA ATENDIDA.

1.c) origem da obtenção da cafeína e do placebo/fornecedor, nome do responsável técnico pela preparação/dispensação dos produtos;

RESPOSTA: "1.c) Origem da obtenção da cafeína e do placebo, nome do responsável técnico pela preparação dos produtos Página 21, Ítem 4.8 Materiais "Cápsulas de cafeína/placebo: As cápsulas serão manipuladas na Farmácia de Manipulação "Farmacotécnica" loja localizada na 316 norte – sendo a farmacêutica responsável Edna Santos de Almeida (CRF/DF: 4.508)"
ANÁLISE: PENDÊNCIA ATENDIDA.

1.d) riscos decorrentes das entrevistas e da realização dos exames em que estão envolvidos equipamentos e técnicas citados no projeto, bem como os procedimentos para evitá-los, minimizá-los e providencias caso ocorram;

RESPOSTA: “1.d) Riscos decorrentes das entrevistas e da realização dos exames em que estão envolvidos equipamentos e técnicas citados no projeto, bem como os procedimentos para evitá-los, minimizá-los e providencias caso ocorram Página 20, Ítem 4.6 Análise dos riscos e benefícios “Além disso, a participação no estudo envolve o desconforto decorrente do tempo para realização dos testes, que é cerca de 3 horas no total. Os exames realizados não são invasivos e não trazem nenhum risco adicional.””
ANÁLISE: PENDÊNCIA ATENDIDA.

1.e) padronização dos critérios de inclusão e exclusão, os documentos apresentados devem conter a mesma informação;

RESPOSTA: “1.e) Padronização dos critérios de inclusão e exclusão, os documentos apresentados devem conter a mesma informação Foram modificados os critérios descritos na plataforma brasil e no Projeto (página 19). Desta forma: “4.4.1. Critérios de Inclusão: Participarão do estudo voluntários que sejam maiores de 18 anos e possuam queixa de zumbido; 4.4.2. Critérios de Exclusão: Serão excluídos do estudo os sujeitos que apresentem zumbido objetivo, zumbido agudo (menos de seis meses de aparecimento do zumbido), que possuam distúrbios psiquiátricos e/ou cognitivos, alteração de orelha média e/ou externa e/ou alguma alergia à cafeína e/ou amido de milho.”
ANÁLISE: PENDÊNCIA ATENDIDA.

1.f) como e quando será oferecido o TCLE ao participante, considerando que a primeira parte da pesquisa consta de questionário on line ("ProjetoCEP.docx", pág. 21/40);

RESPOSTA: “1.f) como e quando será oferecido o TCLE ao participante, considerando que a primeira parte da pesquisa consta de questionário on line (“Projeto CEP.docx”, pág. 21/40) Página 19, Ítem 4.5 Aspectos Éticos “No questionário on line serão disponibilizadas informações quanto aos objetivos e metodologia do estudo, tendo o respondente do questionário a opção de

aceitar ser contatado para mais informações ou não aceitar participar do estudo. Aqueles que se enquadrarem nos critérios de seleção e forem convocados a participar do estudo serão informados sobre a metodologia do mesmo durante o contato telefônico e assinarão o TCLE durante o encontro presencial para realização dos exames, podendo retirar seu consentimento sem prejuízo algum. Os que não se enquadrarem nos critérios de seleção serão contatados via ligação telefônica sendo informados sobre o motivo da exclusão e os que envolverem questões auditivas terão a oportunidade de ser atendidos por um especialista. Os participantes que desejaram deixar de participar da pesquisa em qualquer fase não sofreram nenhum tipo de penalidade.””
ANÁLISE: PENDÊNCIA ATENDIDA.

1.g) a garantia de confidencialidade e sigilo da identificação dos participantes e das informações e dados obtidos;
RESPOSTA: “1.g) A garantia a confidencialidade e sigilo da identificação do participantes e das informações e dados obtidos; Página 19, Ítem 4.5 Aspectos Éticos “Os dados coletados serão veiculados através de artigos científicos, em revistas especializadas e/ou em encontros científicos e congressos, sem nunca tornar possível a identificação dos participantes, assegurando desta forma, o sigilo e a privacidade.””
ANÁLISE: PENDÊNCIA ATENDIDA.

1.h) custo do colaborador da pesquisa e do estatístico ("ProjetoCEP.docx", pág. 22/40) no demonstrativo de custos e a citação de quem será a responsabilidade do custeio, com a incorporação das informações nos documentos "PB_INFORMAÇÕES _BÁSICAS_DO_PROJETO_804358.pdf" e "ProjetoCEP.docx".

RESPOSTA: “1.h) custo do colaborador da pesquisa e do estatístico (Projeto CEP p. 22/40) no demonstrativo de custos e a observação de quem será a responsabilidade do custeio. Plataforma Brasil e no Projeto Página 30, Ítem 6. Orçamento “Estatístico R$500,00, Colaborador voluntário R$0,00” e Página 23, 20 parágrafo “Um colaborador voluntário controlará os participantes que receberão cafeína e os que receberão placebo...””
ANÁLISE: PENDÊNCIA ATENDIDA.

2 – inclusão na Plataforma Brasil do Instituto Brasiliense de Otorrinolaringologia como instituição coparticipante da pesquisa (Res. CNS 466/2012, item II.9).

RESPOSTA: “2. Inclusão na Plataforma Brasil do Instituto Brasiliense de Otorrinolaringologia como instituição coparticipante da pesquisa (Res. CNS 466/2012, item II.9) – Incluído”
ANÁLISE: PENDÊNCIA ATENDIDA.

3 – No TCLE devem constar as seguintes informações (Res. CNS 466/2012, itens II.23, IV.3, b, c, e):

3.a) descrição dos riscos decorrentes da ingestão da cafeína e do placebo, bem como os procedimentos para evitá-los, minimizá-los e providencias caso ocorram;

RESPOSTA: “3.a) descrição dos riscos decorrentes da ingestão da cafeína e do placebo, bem como os procedimentos para evitá-los, minimizá-los e providencias caso ocorram Página 1, 60 parágrafo “A ingestão da cafeína pode gerar aumento do estado de vigília e sensação de alerta, que tem remissão espontânea após o tempo de ação da substância que varia de 2 a 4 horas. O placebo não gera nenhum desconforto.””
ANÁLISE: PENDÊNCIA ATENDIDA.

3.b) descrição dos riscos decorrentes das entrevistas e da realização dos exames em que estão envolvidos equipamentos e técnicas, bem como os procedimentos para evitá-los, minimizá-los e providencias caso ocorram;

RESPOSTA: “3.b) descrição dos riscos decorrentes das entrevistas e da realização dos exames em que estão envolvidos equipamentos e técnicas, bem como os procedimentos para evitá-los, minimizá-los e providencias caso ocorram Página 1, 60 parágrafo “Além disso, a participação no estudo envolve o desconforto decorrente do tempo para realização dos testes, que é cerca de 3 horas no total. Os exames realizados não são invasivos e não trazem nenhum risco adicional.””
ANÁLISE: PENDÊNCIA ATENDIDA.

3.c) inclusão da garantia da confidencialidade e sigilo da identidade do participante e das informações obtidas;

RESPOSTA: “3.c) Inclusão da garantia da confidencialidade e sigilo das informações obtidas

Página 1, 90 parágrafo “Nos comprometemos a utilizar os dados coletados somente para pesquisa, e os resultados serão veiculados através de artigos científicos, em revistas especializadas e/ou em encontros científicos e congressos, sem nunca tornar possível a sua identificação, assegurando desta forma, o sigilo e a privacidade dos seus dados.””
ANÁLISE: PENDÊNCIA ATENDIDA.

3.d) Em complemento a afirmação de que “A sua participação nesta pesquisa é voluntária” deve ser adicionado que não haverá pagamento ou contrapartida pela participação na pesquisa.

RESPOSTA: “3.d) Em complemento a afirmação de que “A sua participação nesta pesquisa é voluntária” deve ser adicionado que não haverá pagamento ou contrapatida pela participação na pesquisa Página 2, 20 parágrafo “Sua participação não implica em pagamento ou qualquer tipo de remuneração ou contrapartida.””
ANÁLISE: PENDÊNCIA ATENDIDA.

3.e) inclusão da informação de que os dados e informações decorrentes da pesquisa estarão à disposição com o pesquisador por cinco anos;

RESPOSTA: “3.e) inclusão da informação de que os dados e informações decorrentes da pesquisa estarão com o pesquisador por cinco anos Página 1, 90 parágrafo “As informações e dados decorrentes da pesquisa ficaram armazenadas com o pesquisador principal por um período de 5 anos.””
ANÁLISE: PENDÊNCIA ATENDIDA.

3.f) inclusão, no espaço destinado a rubrica na primeira página, da identificação de que se trata de rubrica do participante e incluir o espaço para rubrica do pesquisador;
RESPOSTA: “3.f) inclusão, no espaço destinado a rubrica na primeira página, da identificação de que se trata da rubrica do participante e incluir o espaço para rubrica do pesquisador Inclusões realizadas”
ANÁLISE: PENDÊNCIA ATENDIDA.

3.g) correção da numeração das páginas (ex. pág. 1 de 2, pág. 2 de 2), a fim de garantir a integridade do documento.

RESPOSTA: “3.g) correção da numeração das páginas (ex pág 1 de 2, pág 2 de 2), a fim de garantir a integridade do documento Correção feita - Cabeçalho (canto superior direito) de cada página”
ANÁLISE: PENDÊNCIA ATENDIDA.

4 - atualização do cronograma de atividades, em especial, a etapa de coleta de dados, de forma a prever o trâmite do protocolo de pesquisa no CEP/FS.

RESPOSTA: 4. Atualização do cronograma de atividades, em especial, a etapa de coleta de dados, de frma a prever o trâmite do protocolo de pesquisa no CEP/FS Realizado tanto no projeto (pág 29) quanto na Plataforma Brasil

ANÁLISE: O início da coleta de dados – 01 jun 2017. PENDÊNCIA ATENDIDA.

CONCLUSÃO FINAL
Foram atendidas pendências decorrentes da avaliação da Versão 1 incluída na Plataforma Brasil.
O projeto em questão, após avaliação da Versão 2, está de acordo com a Res. 466/2012 e demais normativas pertinentes.

Considerações Finais a critério do CEP:
De acordo com a Resolução 466/12 CNS, itens X.1.- 3.b. e XI.2.d, os pesquisadores responsáveis deverão apresentar relatórios parcial semestral e final do projeto de pesquisa, contados a partir da data de aprovação do protocolo de pesquisa.

Este parecer foi elaborado baseado nos documentos abaixo relacionados:

Tipo Documento	Arquivo	Postagem	Autor	Situação
Informações Básicas do Projeto	PB_INFORMAÇÕES_BÁSICAS_DO_PROJETO_804358.pdf	05/04/2017 08:26:59		Aceito
Outros	cartaResposta.docx	05/04/2017 08:25:24	Alleluia Lima Losno Ledesma	Aceito
Projeto Detalhado / Brochura Investigador	ProjetoCEP2.docx	05/04/2017 08:24:04	Alleluia Lima Losno Ledesma	Aceito
TCLE / Termos de Assentimento / Justificativa de	TCLE2.docx	05/04/2017 08:23:24	Alleluia Lima Losno Ledesma	Aceito

Ausência	TCLE2.docx	05/04/2017 08:23:24	Alleluia Lima Losno Ledesma	Aceito
Outros	TermoConcordCEPFS.doc	14/12/2016 14:10:55	Alleluia Lima Losno Ledesma	Aceito
Outros	termrespcompsemassinatura.docx	14/12/2016 14:07:40	Alleluia Lima Losno Ledesma	Aceito
Outros	cartaencaminhprojeto.doc	14/12/2016 14:06:50	Alleluia Lima Losno Ledesma	Aceito
Outros	curriculofayez.docx	01/12/2016 10:24:12	Alleluia Lima Losno Ledesma	Aceito
Outros	Curriculo.doc	01/12/2016 09:53:51	Alleluia Lima Losno Ledesma	Aceito
Outros	TermoConcord.jpg	01/12/2016 09:47:42	Alleluia Lima Losno Ledesma	Aceito
Outros	TermoRespCompromPesqCEPFS.doc	01/12/2016 09:43:11	Alleluia Lima Losno Ledesma	Aceito
Outros	CartaEncaminhamento.jpg	01/12/2016 09:39:21	Alleluia Lima Losno Ledesma	Aceito
Folha de Rosto	Folha_rosto.pdf	24/10/2016 21:20:10	Alleluia Lima Losno Ledesma	Aceito

Situação do Parecer:
Aprovado

Necessita Apreciação da CONEP:
Não

BRASILIA, 25 de Abril de 2017

Assinado por:
Keila Elizabeth Fontana
(Coordenador)
