# Supplementary material for: The effect of caffeine on tinnitus: Randomized triple-blind placebo-controlled clinical trial
Source: PLoS One. 2021 Sep 20;16(9):e0256275. doi: 10.1371/journal.pone.0256275 (PMC8452027; doi:10.1371/journal.pone.0256275)
Supplement: S3 Appendix — (DOCX) [file pone.0256275.s004.docx]

UNB - FACULTY OF HEALTH SCIENCES OF THE UNIVERSITY OF BRASÍLIA

CONSUBSTANCED OPINION OF THE INSTITUTIONAL RESEARCH BOARD

RESEARCH PROJECT DATA

Research Title: The effect of caffeine on self-perception of tinnitus

Researcher: Alleluia Lima Losno Ledesma

Thematic Area:

Version: 2

CAAE: 63290116.2.0000.0030

Proponent Institution: Faculty of Health Sciences, University of Brasília

Main Sponsor: Own Financing

OPINION DATA

Opinion Number: 2,031,285

PROJECT PRESENTATION

"Abstract:

Introduction: Some authors suggest that, because caffeine is a central nervous system stimulant, its use can cause a change in the self-perception of tinnitus. At the same time, clinical experience suggests that a diet that restricts the use of caffeine does not modify the patient's discomfort in relation to tinnitus, which can lead to increased discomfort and decreased patient collaboration with medical recommendations. Until a consensus is reached, each service adopts the recommendations that seem convenient, since in the literature the topic is still controversial, therefore, it does not bring definitions about the influence of this substance on the tinnitus complaint. Objectives: To analyze the influence of caffeine on self-perception of tinnitus. Methods: Eighty young and healthy individuals will be selected, with no other auditory or vestibular complaints, except tinnitus. The subjects will be randomly divided into two groups: caffeine and placebo. A diet that restricted caffeine consumption for 24 hours was implemented and the individuals will be submitted to exams and questionnaires in two timepoints: at baseline and after capsule ingestion**.** "

“Introduction: Tinnitus is defined as an auditory perception in the absence of an external sound source (Coelho et al., 2004; Almeida et al., 2009). According to the World Health Organization (WHO), 278 million people have tinnitus, approximately 15% of the world population. This prevalence increases to 33% among individuals over 60 years of age (Jastebroff, 1980; Coelho et al., 2004; Pinto et al., 2010). In Brazil, it is estimated that more than 28 million individuals have tinnitus, making it a public health problem (Sanchez et al., 2002). Caffeine (1, 3, 7-trimethylxanthine) is a central nervous system stimulant belonging to the group of methylxanthines (CAMARGO and TOLEDO, 1998; MYCEK, HARVEY and CHAMPE, 1998; SILVA, 1998; KATZUNG, 2005; ALVES, CASAL and OLIVEIRA, 2009). Its metabolism has been studied since 1850, even before its chemical characterization (1895), and even today there are no definitive conclusions (CAMARGO, 1996). Because caffeine is a central stimulant, some authors suggest that its use may cause a change in the self-perception of tinnitus. At the same time, clinical experience suggests that a diet that restricts the use of caffeine does not modify the patient's discomfort in relation to tinnitus, which can lead to increased discomfort and decreased patient collaboration with medical recommendations. Until a consensus is reached, each service adopts the recommendations that seem convenient, since in the literature the topic is still controversial, therefore, it does not bring definitions about the influence of this substance on the tinnitus complaint. ”

"Hypothesis: Caffeine does not influence self-perception of tinnitus"

“Proposed Methodology:

Participants will answer an online questionnaire seeking to identify those who meet the study selection criteria and the Food Habits Questionnaire (adapted from Camargo 1996) seeking to know the amount of caffeine consumed by the participants daily. From the information obtained in the Food Habits Questionnaire, the amount of caffeine consumed daily by the participants will be calculated, using the values ​​found in the study by Camargo (1996) regarding the volume of the containers and the caffeine content of each product. The participants will then be classified according to the amount of caffeine they usually consume daily, as proposed by Schreiber et al. (1988): sporadic consumption (less than 100mg), light (from 100 to 299mg), moderate (300 to 499mg). Those with consumption greater than or equal to 500mg / day (intense or very intense) will not participate in the other stages of the study. Those participants who do not meet the selection criteria will have their doubts clarified and the reasons for exclusion will be explained to them. When the reasons for exclusion involve auditory and / or vestibular complaints, participants will be instructed to seek an ENT doctor for a detailed assessment. Those who meet the selection criteria will be advised to abstain from foods / products containing caffeine 24 hours before participating in the study. Upon arriving at the clinic, participants will be asked about compliance with the recommendations for the exams and those who declare they have not followed, however, still wish to participate in the study, will have another meeting scheduled. Those who have followed the recommendations will respond to the POMS, THI and EVA, in the order described, then pure tone audiometry, acuphenometry and Distortion Product Otoacoustic Emissions will be performed. After this first moment, the participants received capsules that can contain placebo or caffeine (300mg) with a small amount of water. One hour after ingestion, they will respond again to POMS, THI and EVA and will be submitted again to acuphenometry and Distortion Product Otoacoustic Emissions. A study contributor will control participants who will receive caffeine and those who will receive placebo, with the participants, researcher and statistician not being informed which participants belong to each group. ”

Inclusion Criteria: "Volunteers who are over 18 and have tinnitus complaints will participate in the study."

Exclusion Criterion: “Subjects who have metabolic changes, hormonal changes, psychic disorders, neurological diseases, who use drugs for continuous use, smokers, alcoholics and users of illicit drugs will be excluded from the study. "

“Data Analysis Methodology: Measurements at baseline, in both groups, will be compared using the Chi-square test or Fisher's exact test (in the case of expected frequencies less than 1) for qualitative variables. In the case of quantitative variables with Gaussian distribution, in both groups, the Student t test, or the non-parametric Mann-Whitney test, will be used for those without Gaussian distribution. Post-intervention values of variables (acuphenometry) will be compared between groups using a covariance analysis model (ANCOVA). These values will be considered, in the statistical model, as a dependent variable, the type of intervention will be the independent variable and the values at the baseline of the variables will be considered as a covariate. P <0.05 will be considered significant. The analysis will be performed using the SAS 9.4 application (SAS Institute, Inc., 1999). ”

“Primary outcome:

The scores of the acuphenometry results will be compared before and after the use of caffeine / placebo in order to identify the general impression of the discomfort related to tinnitus in the participants at both times.

Secondary outcome:

Analyze the scores before and after the use of caffeine / placebo in each of the exams and questionnaires separately, observing which aspects suffered or not influenced by the substance “Sample Size in Brazil: 80”

Research objectives

“Primary Objective: To analyze the influence of caffeine on self-perception of tinnitus.

Secondary Objective:

Analyze the influence of caffeine on acuphenometry

Analyze the influence of caffeine on the Tinnitus Handicap Inventory (THI)

Analyze the influence of caffeine on Visual Analogue Scale (VAS)

Analyze the influence of caffeine on Distortion product otoacoustic emissions (DPOAEs)

Analyze changes in mood before the exam.”

Assessment of risks and benefits

"Risks:

The risks inherent in the study are common in caffeine withdrawal: headache, fatigue, lethargy, “flu-like symptoms” and mood disorders. These symptoms are temporary and easily controlled by analgesics. Furthermore, the individual must have time to perform the testing, which is about 3 hours in total.

Benefits:

As benefits, the individual will have the opportunity to have an assessment of the auditory system, and is oriented on the bahavior thereof, and to be attended by a specialist if there is change in the conventional exam (with restriction of caffeine). "

Research Comments and Considerations

This is a project by researcher Alleluia Lima Losno Ledesma, from the Post-Graduation Program in Health Sciences at the Faculty of Health Sciences at UnB, under the guidance of Dr. Fayez Bahmad Junior.

Place of research: Instituto Brasiliense de Otorrinolaringologia.

Main sponsor: Own financing, using equipment from the place where the project is carried out.

Considerations on the Mandatory Submission Terms:

PB_INFORMAÇÕES_BÁSICAS_DO_PROJETO_804358.pdf - attached on Apr 05, 2017 - project with basic information, with modifications in the text in order to meet the pending issues mentioned in the previous version analysis, as well as changes in the Execution and Budget Schedule Financial. cartaResposta.docx - appended on Apr 05, 2017 - letter replying to CEP / FS, undated and unsigned, with the description of the changes that were carried out, or the complementation of information, in order to meet the pending issues pointed out in the analysis of the previous version . ProjetoCEP2.docx - annexed on Apr 5, 2017 - complete project presented with the necessary modifications and additions to meet the pending issues verified in the analysis of the previous version. TCLE2.docx - attached on Apr 05, 2017 - document modified in response to the pending issues verified in the analysis of the previous version.

Recommendations: Not applicable.

Conclusions or Pending and List of Inadequacies:

The issues identified by Consubstantiated Opinion No. 1,962,542, of March 13, 2017, were verified:

1 - the documents "ProjetoCEP.docx" and the "PB_INFORMAÇÕES_BÁSICAS_DO_ PROJETO_804358.pdf" must contain the information (Res. CNS 466/2012, items II.3; II.10; II.22; III.1, d; III. 2 a, b; IV.1 b; IV.3 e): 1.a) risks associated with caffeine ingestion, as well as the procedures to avoid them, minimize them and measures in case they occur, as well as the dosage justification to be offered to the research participant;

ANSWER: “1.a) Risks associated with caffeine intake, as well as the procedures to avoid them, minimize them and measures in case they occur, as well as the justification of the dosage to be offered to the research participant; Page 20, Item 4.6 Analysis of risks and benefits “Caffeine intake (300mg) can cause an increase in the state of wakefulness and a feeling of alertness, which spontaneously remits after the time of action of the substance, which varies from 2 to 4 hours. This concentration refers to a moderate consumption of caffeine, the average consumption being usual in Brazilians. "

ANALYSIS: PENDING ATTENDED.

1.b) description of the placebo to be used and dosage, the risks associated with its ingestion, as well as the procedures to avoid them, minimize them and measures in case they occur;

ANSWER: “1.b) Description of the placebo to be used and dosage, the risks associated with its ingestion, as well as the procedures to avoid them, minimize them and measures if they occur Page 20, Item 4.6 Analysis of risks and benefits“ The placebo used will be corn starch (1g), and its intake does not cause any discomfort in people who are not allergic to the substance. ”

ANALYSIS: PENDING ATTENDED.

1.c) origin of obtaining caffeine and placebo / supplier, name of the technician responsible for preparing / dispensing the products;

ANSWER: “1.c) Origin of obtaining caffeine and placebo, name of the technician responsible for preparing the products Page 21, Item 4.8 Materials“ Caffeine capsules / placebo: The capsules will be handled at the “Farmacotécnica Manipulation Pharmacy” store located at 316 Norte - with the pharmacist in charge Edna Santos de Almeida (CRF / DF: 4.508) ”

ANALYSIS: PENDING ATTENDED.

1.d) risks arising from interviews and perform tests involved equipment and techniques cited in the project, as well as procedures to avoid them, minimize them and providences should they occur

ANSWER: "1.d) risks arising from interviews and clinical exams that are involved equipment and techniques cited in the project, as well as procedures to avoid them, minimize them and if they occur providences Page 20, Item 4.6 Analysis of risks and benefits “In addition, participation in the study involves the discomfort resulting from the time to perform the tests, which is about 3 hours in total. The tests performed are non-invasive and do not pose any additional risk. ”

ANALYSIS: PENDING ATTENDED.

1.e) standardization of the inclusion and exclusion criteria, the documents presented must contain the same information;

ANSWER: “1.e) Standardization of the inclusion and exclusion criteria, the documents presented must contain the same information The criteria described in the brasil platform and in the Project have been modified (page 19). In this way: “4.4.1. Inclusion Criteria: Volunteers who are over 18 and have tinnitus complaints will participate in the study; 4.4.2. Exclusion Criteria: Subjects who present objective tinnitus, acute tinnitus (less than six months after the onset of tinnitus), who have psychiatric and / or cognitive disorders, alteration of the middle and / or external ear and / or any allergy will be excluded from the study. to caffeine and / or corn starch. ”

ANALYSIS: PENDING ATTENDED.

1.f) How and when the written informed consent will be offered to the participant, considering that the first part of the research consists of an online questionnaire ("ProjetoCEP.docx", page 21/40);

ANSWER: “1.f) how and when will the written informed consent be offered to the participant, considering that the first part of the research consists of an online questionnaire (“ CEP.docx Project ”, page 21/40) Page 19, Item 4.5 Ethical Aspects “The online questionnaire will provide information on the study's objectives and methodology, with the questionnaire respondent having the option to accept being contacted for more information or not accepting to participate in the study. Those who meet the selection criteria and are invited to participate in the study will be informed about the methodology of the study during telephone contact and will sign the informed consent form during the face-to-face meeting for the exams, being able to withdraw their consent without prejudice. Those who do not meet the selection criteria will be contacted via phone call, being informed about the reason for the exclusion and those that involve hearing issues will have the opportunity to be attended to by a specialist. The participants who wished to stop participating in the research at any stage did not suffer any type of penalty. ”

ANALYSIS: PENDING ATTENDED.

1.g) the guarantee of confidentiality and secrecy of the identification of the participants and of the information and data obtained;

ANSWER: “1.g) Guarantee the confidentiality and secrecy of the identification of the participants and the information and data obtained; Page 19, Item 4.5 Ethical Aspects “The data collected will be conveyed through scientific articles, in specialized magazines and / or at scientific meetings and congresses, without ever making it possible to identify the participants, thus ensuring secrecy and privacy.”

ANALYSIS: PENDING ATTENDED.

1.h) cost of the research collaborator and the statistician ("ProjetoCEP.docx", page 22/40) in the cost sheet and the specification of who will be responsible for the cost, with the incorporation of the information in the documents "PB_INFORMAÇÕES _BÁSICAS_DO_PROJETO_804358 .pdf "and" ProjetoCEP.docx ".

ANSWER: “1.h) cost of the research collaborator and the statistician (Project CEP p. 22/40) in the cost statement and the observation of who will be responsible for the cost. Plataforma Brasil and Project Page 30, Item 6. Budget “Statistical R $ 500.00, Voluntary employee R $ 0.00” and Page 23, 20 paragraph “A volunteer employee will control participants who will receive caffeine and those who will receive placebo ... ”

ANALYSIS: PENDING ATTENDED.

2 - inclusion in the Brasil Platform of the Brasiliense Institute of Otorhinolaryngology as a co-participant institution in the research (Res. CNS 466/2012, item II.9).

ANSWER: “2. Inclusion in the Brasil Platform of the Brasiliense Institute of Otorhinolaryngology as a co-participant institution in the research (Res. CNS 466/2012, item II.9) - Included ”

ANALYSIS: PENDING ATTENDED.

3 - In the written informed consent, the following information must be included (Res. CNS 466/2012, items II.23, IV.3, b, c, e):

3rd) description of the risks resulting from the intake of caffeine and placebo, as well as procedures to avoid them, minimize them and providences should they occur; ANSWER: "3.a) description of the risks resulting from the intake of caffeine and placebo, as well as procedures to avoid them, minimize them and if they occur providences page 1, paragraph 60" intake of caffeine can lead to increased state of wakefulness and a feeling of alertness, which has spontaneous remission after the time of action of the substance that varies from 2 to 4 hours. The placebo did not generate any discomfort. "

ANALYSIS: PENDING ATTENDED.

3.b) description of the risks arising from the interviews and from the examinations in which equipment and techniques are involved, as well as the procedures to avoid them, minimize them and measures in case they occur;

ANSWER: “3.b) description of the risks arising from the interviews and the examinations in which equipment and techniques are involved, as well as the procedures to avoid them, minimize them and take measures in case they occur Page 1, 60 paragraph“ In addition , participation in the study involves discomfort from time to perform the test, which is about three hours in total. The examinations are noninvasive and do not bring any additional risk. "

ANALYSIS: PENDING ATTENDED.

3.c) inclusion of the guarantee of confidentiality and secrecy of the participant's identity and the information obtained;

ANSWER: "3c) Inclusion of the guarantee of confidentiality and secrecy of the information obtained page 1, paragraph 90" We commit ourselves to use the collected data only for research, and the results will be conveyed through scientific articles in journals and / or in scientific conferences and meetings, never allow their identification, thus ensuring the confidentiality and privacy of your data. ""

ANALYSIS: PENDING ATTENDED.

3.d) In addition to the statement that “Your participation in this research is voluntary” must be added that there will be no payment or compensation for participation in the research.

ANSWER: “3.d) In addition to the statement that“ Your participation in this survey is voluntary ”must be added that there will be no payment or compensation for participation in the survey Page 2, 20 paragraph“ Your participation does not imply payment or any type remuneration or compensation. ""

ANALYSIS: PENDING ATTENDED.

3.e) inclusion of information that the data and information resulting from the research will be available to the researcher for five years;

ANSWER: “3.e) inclusion of information that the data and information resulting from the research will be with the researcher for five years Page 1, 90 paragraph“ The information and data resulting from the research were stored with the main researcher for a period of 5 years.""

ANALYSIS: PENDING ATTENDED.

3.f) inclusion in the space for the heading on the first page, identifying it is the participant's item and include space for entry of the researcher;

ANSWER: "3.f) inclusion in the space for the heading on the first page, identifying it is the participant's item and include space for inclusions researcher at the line pass"

ANALYSIS: PENDING ATTENDED.

3.g) correction of page numbering (eg, page 1 of 2, page 2 of 2), in order to guarantee the integrity of the document.

ANSWER: "3.g) correction of the number of pages (eg page 1 of 2 page 2 of 2) in order to ensure document integrity correction made - Header (top right) of each page"

ANALYSIS: PENDING ATTENDED.

4 - update of the activity schedule, in particular, the data collection stage, in order to provide for the processing of the research protocol at CEP / FS.

ANSWER: 4. Update of the activity schedule, in particular, the data collection stage, in order to provide for the processing of the research protocol at CEP / FS Performed both in the project (page 29) and in the Brazil Platform. The beginning of data collection - 01 Jun 2017.

ANALYSIS: PENDING ATTENDED.

FINAL CONCLUSION

Pending issues resulting from the evaluation version initially included in the Brazil Platform were answered. The project in question, after evaluation of Version 2, is in accordance with Res. 466/2012 and other pertinent regulations.

Final Considerations at the discretion of the CEP:

According to CNS Resolution 466/12, items X.1.- 3.b. and XI.2.d, the researchers in charge must submit partial half-yearly and final reports of the research project, counted from the date of approval of the research protocol.

This opinion was prepared based on the documents listed below:

| **Document Type** | **File** | **Post** | **Author** |
| --- | --- | --- | --- |
| Basic information  from the project | PB_INFORMAÇÕES_BÁSICAS_DO_P ROJETO_804358.pdf | 05/04/2017 08:26:59 | Alleluia Lima Losno Ledesma |
| Others | cartaResposta.docx | 05/04/2017 08:25:24 | Alleluia Lima Losno Ledesma |
| Detailed project /  Brochure  Investigator | ProjetoCEP2.docx | 05/04/2017 08:24:04 | Alleluia Lima Losno Ledesma |
| TCLE/ Terms of  Nod /  Justification of | TCLE2.docx | 05/04/2017 08:23:24 | Alleluia Lima Losno Ledesma |
| Absence | TCLE2.docx | 05/04/2017 08:23:24 | Alleluia Lima Losno Ledesma |
| Others | TermoConcordCEPFS.doc | 14/12/2016 14:10:55 | Alleluia Lima Losno Ledesma |
| Others | termrespcompsemassinatura.docx | 14/12/2016 14:07:40 | Alleluia Lima Losno Ledesma |
| Others | cartaencaminhprojeto.doc | 14/12/2016 14:06:50 | Alleluia Lima Losno Ledesma |
| Others | curriculofayez.docx | 01/12/2016 10:24:12 | Alleluia Lima Losno Ledesma |
| Others | Curriculo.doc | 01/12/2016 09:53:51 | Alleluia Lima Losno Ledesma |
| Others | TermoConcord.jpg | 01/12/2016 09:47:42 | Alleluia Lima Losno Ledesma |
| Others | TermoRespCompromPesqCEPFS.doc | 01/12/2016 09:43:11 | Alleluia Lima Losno Ledesma |
| Others | CartaEncaminhamento.jpg | 01/12/2016 09:39:21 | Alleluia Lima Losno Ledesma |
| Title page | Folha_rosto.pdf | 24/10/2016 21:20:10 | Alleluia Lima Losno Ledesma |

Opinion Status: Approved

Needs CONEP Appreciation: No

BRASILIA, April 25, 2017

Signed by:

Keila Elizabeth Fontana

(Coordinator)
