# Supplementary material for: The effect of caffeine on tinnitus: Randomized triple-blind placebo-controlled clinical trial
Source: PLoS One. 2021 Sep 20;16(9):e0256275. doi: 10.1371/journal.pone.0256275 (PMC8452027; doi:10.1371/journal.pone.0256275)
Supplement: Attachment — Submitted filename: Response to Reviewers.docx [file pone.0256275.s005.docx]

Response to Reviewers

I would like to thank the considerations made regarding the submitted manuscript. They were taken into account and certainly added quality to the study. Below are the answers to each consideration made.

- The naming of the files has been formatted according to the journal's guidelines
- The recruitment method as well as the recruitment and data collection location was further detailed in the text as requested: Chapter Methods, subtopics "Recruitment" (page 5, lines 89-90) and "Procedures" (page 7, lines 133-134)
- I would like to apologize for the mistake in the article. There was an error writing the foreign language. Registration was made on the REBEC platform in March, being accepted in July. Recruitment started shortly after being "accepted" in July (not in June as previously described in the article): Chapter Methods, subtopics "Recruitment" (page 5, line 91)
- The requested declaration “The authors confirm that all ongoing and related trials for this drug / intervention are registered” has been added in the chapter "Methods" subtopic "Study design, approvals, Registration and Patient Consents" (page 4, line 78-79).
- The full name of the institutional review board has been added in the "Methods" subsection "Study design, approvals, Registration and Patient Consents" chapter (page 4, line 75), as well as in the submission form
- The corresponding author's ORCID ID was added in Editorial Manager
- The caption for figure 2 has been included: page 9, line 195
- Sample size calculation: The description of the sample size calculation has been reformulated in order to make it clearer (Chapter “Methods” subtopic “Statistical analysis”, page 9, lines 174-178).
  - For the sample calculation, the THI results were considered as the primary outcome
  - The sample size considering that non-parametric tests were used was determined by multiplying the sample size calculated for parmetric tests by a correction factor. This correction factor is ARE (Asymptotic Relative Efficiency), described by Pitman. To calculate the sample size, α equal to 0.05 was used, test power (1-β) equal to 0.8, effect equal to 0.5, using the conservative ARE minimum method. The ARE method reference was: Lehmannn, EL. Nonparametrics: Statistical methods based on ranks. San Francisco, CA: Holden-Day, 1975; Pitaman, E.J.G, (1948), Lecture notes on nonparametric statistical inference, Columbia University.
- The description of the calculation of the amount of habitual consumption of caffeine has been included, as well as its reference: Chapter “Methods” subtopic “Questionnaires and exams”, page 7, lines 137-141 and reference [20].
- The description of  how the participants were instructed to restrict caffeine consumption for 24 hours preceding the intervention has been included: Chapter “Methods” subtopic “Procedures”, page 6/7, lines 128-130.
- The subtopic "The active ingredient" was added in chapter “Methods” in order to explain the choice of measures 1 hour after caffeine consumption based on its chemical effects (page 5/6, line 107-111) and the choice of 300mg dose (page 6, line 112-118).
- Table 1:
  - Range values have been replaced by IQRs (page 10)
- Table 2:
  - In order to allow the correct interpretation of the values presented in "Table 2", the description of the tests used (THI and VAS) was added in the chapter "Methods", sub item "Questionnaires and exams" (page 7, lines 143-144), as well as their references [25,26]
  - The title of the table has been adjusted to show that it represents the correlation between caffeine consumption in the usual diet and the characteristics described in the same table (page 11)
  - An error occurred in the formatting and layout of the "*" signs. They were reorganized and presented median and interquartile ranges (pages 11/12)
- The "Discussion" chapter has been revised to make it more compact and cohesive in order not to wander from one point to another (pages 14-18). Because of this, a revision of the "Introduction" chapter was necessary (page 3)
  - Page 14, lines 276-280: The first paragraph of the discussion was structured in order to verify whether our sample was similar to the profile of patients with tinnitus reported in studies conducted in this population previously regarding sex, age, duration and location of tinnitus and discomfort in relation to it (calculated with the same instrument used in this study).
  - The studies to which ours was compared were better described, as proposed, in order to make this comparison clearer: page 16, lines 306-312 and 321-323. A comparison was made between the values found in the VAS in our study with the values found in the only two studies that investigated the relationship between caffeine and tinnitus and that used the same instrument. One of these studies was cross-sectional and retrospectively investigated in 107 individuals whether there was a relationship between the discomfort with tinnitus (assessed by VAS) and the consumption of caffeine in the usual diet. This relationship is not observed. The other study assessed the influence of long-term caffeine reduction on VAS results. Our study was compared to previous studies that presented the pitch and loudness of tinnitus in a sample of adult individuals, without, however, relating these measures to caffeine consumption. These studies were chosen as the studies that linked caffeine to tinnitus showed no such measures
  - Some inferences have been restructured to make them more cautious, as recommended: page 16, lines 316-317; page 17, lines 336/339
  - Observations on the limitations of the study were added in order to justify the choice of the procedures cited for the study methodology: page 18, lines 351-353
