# Supplementary material for: The effect of caffeine on tinnitus: Randomized triple-blind placebo-controlled clinical trial
Source: PLoS One. 2021 Sep 20;16(9):e0256275. doi: 10.1371/journal.pone.0256275 (PMC8452027; doi:10.1371/journal.pone.0256275)
Supplement: Attachment — Submitted filename: Review caffeine.docx [file pone.0256275.s006.docx]

Line 21

Define what the placebo is in the abstract.

Line 26.

24 hours does not seem long as control…? Explain what this is.

Line 27.

VAS here and elsewhere

I think by VAS you mean magnitude estimation. In psychophysics when you are trying to estimate the magnitude of something, it is referred to as Magnitude Estimation. There are several psychophysical procedures that can be used to estimate the magnitude. VAS is one. If one is going to use a VAS scale, it is necessary to state the length of the line, what markings and labels are on the line, and what resolution will be used to convert the subjects marking into a number.

I prefer to have subjects provide a number from 0 to 100.

Line 29

Most readers will not know what acufenometry is.

Line 35

How do you know what is clinically relevant?

Line 48

Please cite the Psychological Model, proposed by Tyler, Aran and Dauman (1992), suggested that the overall annoyance of the tinnitus was a result of the 1) tinnitus characteristics and the 2) psychological make up of each individual patient. Several parts of the brain will be involved in the representation of the tinnitus and of the reactions to the tinnitus. Treatments can be focused on reducing the tinnitus (e.g. pills) or on reducing the reactions to the tinnitus (eg. Counseling )

Tyler, R. S., Aran, J-M., & Dauman, R. (1992). Recent advances in tinnitus. **Am J Audiol**, **1**(4): 36-44.

Line 52

The THI actually uses a 3 label category scale. (SS Stevens.. often referred to as the father of human psychophysics)

Please discuss in the results, the sensitivity of the THI has been challenged.

Tyler, R.S., Oleson, J., Noble, W., Coelho, C., Ji, H. (2007). Clinical trials for tinnitus: Study populations, designs, measurement variables, and data analysis. **Progress in Brain Research, 166:** 499-509.

Line 53

VAS

I think by VAS you mean magnitude estimation. In psychophysics when you are trying to estimate the magnitude of something, it is referred to as Magnitude Estimation. There are several psychophysical procedures that can be used to estimate the magnitude. VAS is one. If one is going to use a VAS scale, it is necessary to state the length of the line, what markings and labels are on the line, and what resolution will be used to convert the subjects marking into a number.

I prefer to have subjects provide a number from 0 to 100.

Line 60

Measuring tinnitus

The dB sensation level is not a direct measure of loudness.

Please reference

Tyler, R. S. & Conrad‑Armes, D. (1983). The determination of tinnitus loudness considering the effects of recruitment. **Journal of Speech and Hearing Research, 26**(1): 59‑72.

You should also cite:

Tyler, R. S., Haskell, G., Gogle, S., & Gehringer, A. (2008). Establishing a Tinnitus Clinic in Your Practice. **Am J Audiol**; 17: 25-37.

They highlighted 4 reasons for measuring tinnitus.

1. Confirm to the patient that their tinnitus is real
2. Monitor changes
3. Provide insights into mechanisms
4. Aid in the fitting of some devices

Tyler, R. S. (1985). Psychoacoustical measurement of tinnitus for treatment evaluations. In: E. Myers (Ed.), **New Dimensions in Otorhinolaryngology‑Head and Neck** **Surgery** (455-458). Amsterdam: Elsevier Publishing Co.

Tyler, R. S. (1992). The psychophysical measurement of tinnitus. In: J-M. Aran & R. Dauman (Eds.), **Tinnitus 91 - Proceedings of the Fourth International Tinnitus Seminar** (17-26). Amsterdam: Kugler Publications.

Tyler, R. S. (2000). The psychoacoustical measurement of tinnitus. In R.S. Tyler (Ed.), **Tinnitus Handbook** (149-179). San Diego, CA: Singular Publishing Group.

Tyler, R. S., Oleson, J., Noble, W., Coelho, C., & Ji, H. (2007). Clinical trials for tinnitus: Study populations, designs, measurement variables, and data analysis. **Progress in Brain Research, 166:** 499-509.

Line 132

Define the duration of the phases.

Line 140

Mate ??

Line 154

Define the protocols so that some can replicate your study, a fundamental of science.

Line 197

Not clear what the 6 month period stands for.

Line 283

Normal hearing..

State how you define this

Normal pure tone thresholds (=< 25 dBHL)

317. changes over time

Please cite Tyler and Baker, who first documented the wide range of problems experienced by tinnitus sufferers and showed that for most patients, the first 6-9 months is the worst.

Tyler, R.S. and Baker, L.J. (1983). Difficulties experienced by tinnitus sufferers. **Journal of Speech and Hearing Disorders, 48**(2): 150‑154.

Line 320

300 Hz. Are you sure??

Please cite Pan et al., whose pitch matching data suggests that most patients do not have a pitch match frequency just below the maximum hearing threshold loss frequency. The argues against the brain reorganization model in most subgroups of tinnitus.

Pan, T., Tyler, R. S., Ji, H., Coelho, C., Gehringer, A., & Gogel, S. (2009). The relationship between tinnitus pitch and the audiogram. **Int J Audiol.** 48 (4): 277-294.

Line 327

dB is not a scale of loudness

Tinnitus sensation level is not a measure of loudness. You might want to consider referencing:

Tyler, R. S. & Conrad‑Armes, D. (1983). The determination of tinnitus loudness considering the effects of recruitment. **Journal of Speech and Hearing Research, 26**(1): 59‑72.

# Abstract

In general, the abstract does not follow conventional policy. The introduction should provide a rationale. The results should provide numeric findings and significance. You might want to view, as an example, guidelines of the American Psychological Association- Publication Manual.

# Acceptance

We talk about control, defeat and acceptance in (please cite)

Tyler, R. S., Coelho, C., & Noble, W. (2006). Tinnitus: Standard of Care, Personality Differences, Genetic Factors. **Journal for Oto-Rhino-Laryngology and its Related Specialities** 68(1):14-22.

# Acronyms

I suggest you limit acronyms , as it will only make the article more difficult to read.

# Attention

G.P. Jacobson, J.A. Calder, C.W. Newman, E.L. Peterson, J.A. Wharton, B.K. Ahmad, Electrophysiological indices of selective auditory attention in subjects with and without tinnitus. Hearing Research, 97, 66-74 (1996).

# Auditory Processing Disorders

Initial attempts to emphasize hearing difficulties not related to the audiogram.

Please cite.

Tyler, R. S. (1976). Temporal integration and cochlear hearing loss. Human Communication, 1: 9 24.

Tyler, R. S., Summerfield, A. Q., Wood, E. J., & Fernandes, M. (1982). Psychoacoustic and phonetic temporal processing in normal and hearing impaired listeners. Journal of the Acoustical Society of America, 72(3): 740 752.

# Bentler

Bentler, R. A., & Tyler, R. S. (1987). Tinnitus management. ASHA, 29(5): 27 32.

# Children

My main concern is that the review of references on research on tinnitus in children is very limited. There are many other important works.

You might want to consider referencing:

| 125 | Stouffer, J. L., Tyler, R. S., Booth, J. C. & Buckrell, B. (1992). Tinnitus in normal-hearing and hearing-impaired children. In: J-M. Aran & R. Dauman (Eds.), **Tinnitus 91 - Proceedings of the Fourth International Tinnitus Seminar** (255-258). Amsterdam: Kugler Publications. |
| --- | --- |

| 184 | Tyler, R. S. & Smith, R. J. (2002). Management of tinnitus in children. In V.E. Newton (Ed.), **Paediatric Audiological Medicine** (397-404). Philadelphia: Whurr Publishers. |
| --- | --- |

| 233 | Coelho, C., Sanchez, T. G., & Tyler, R. S. (2007). Tinnitus in children and associated risk factors. **Progress in Brain Research, 166:** 179-194. |
| --- | --- |

| 264 | Coelho, CB, & Tyler RS (2009) Management of tinnitus in children. Pediatric Audiological Medicine, Second Edition. 21:418-427. |
| --- | --- |

# Children – hearing loss or tinnitus

Please cite an early study attempting to separate hearing loss from tinnitus, in children

Stouffer, J. L., Tyler, R. S., Booth, J. C. & Buckrell, B. (1992). Tinnitus in normal-hearing and hearing-impaired children. In: J-M. Aran & R. Dauman (Eds.), **Tinnitus 91 - Proceedings of the Fourth International Tinnitus Seminar** (255-258). Amsterdam: Kugler Publications.

You could also site references for treating tinnitus in children

e.g.

Tyler, R. S. & Smith, R. J. (2002). Management of tinnitus in children. In V.E. Newton (Ed.), **Paediatric Audiological Medicine** (397-404). Philadelphia: Whurr Publishers.

Kentish, R.C. & Crocker, S.R. (2006) Scary monsters and waterfalls: tinnitus narrative therapy	for children. In: Tyler, R.S. (Ed.), Tinnitus Treatment Clinical Protocols (16) (pp. 217-229). New York: Thieme

# CBT for Tinnitus

CBT uses a very specific orderly protocol in psychological counseling. It has been adapted for many problems.

The first application of CBT for tinnitus was described by Sweetow (in Tyler, R. S. (Ed.). (2000). **Tinnitus Handbook**. San Diego, CA: Singular Publishing Group.

And by Henry and Wilson in their 2 books focused on Tinnitus

Unless the CBT for counseling follows a very formal protocol (such as by Henry and Wilson), the actual benefit depends mostly on the clinician.

The psychological literature has recently noted how CBT can be over-emphasized (see article sited in Tyler, R. S., Mohr, A. M. (2017). Is CBT for Tinnitus Overemphasized? The **Hearing Journal**, Journal Club 70(2):8,10, February 2017. )

CBT principals and exercises were included in Tinnitus Activities Treatment

# CI tinnitus round window

Please cite Kuk et al., who performed round window tinnitus suppression studies.

Kuk, F.K., Tyler, R.S., Rustad, N., Harker, L.A., & Tye‑Murray, N. (1989). Alternating current at the eardrum for tinnitus reduction. **Journal of Speech and** **Hearing Research**, **32**(2): 393‑400.

# CI tinnitus theory

Please reference Tyler et al., 2008 where the different theories are reviewed.

Tyler, R. S., Rubinstein, J., Pan, T., Chang, S., Gogel, S., Gehringer, A., & Coelho, C. (2008). Electrical Stimulation of the Cochlea to Reduce Tinnitus. **Seminars in Hearing.** 29(4): 326-332.

# Cima et al

The Cima et al article had a variety of treatments, and CBT might have had nothing to do the outcome.

# Clinical Trials

Please cite strategies for clinical trials for tinnitus.

Tyler, R. S., Noble, W. G., & Coelho, C. (2006). Considerations for the Design of Clinical Trials for Tinnitus. **Acta Oto-Laryngologica, 126:** 44-49.

Tyler, R. S., Oleson, J., Noble, W., Coelho, C., & Ji, H. (2007). Clinical trials for tinnitus: Study populations, designs, measurement variables, and data analysis. **Progress in Brain Research, 166:** 499-509.

# clinically significant reduction

I have challenged the concepts of “clinically significant reduction”

Please cite

Tyler, R. S., Noble, W. G., & Coelho, C. (2006). Considerations for the Design of Clinical Trials for Tinnitus. **Acta Oto-Laryngologica, 126:** 44-49.

Tyler, R. S., Oleson, J., Noble, W., Coelho, C., & Ji, H. (2007). Clinical trials for tinnitus: Study populations, designs, measurement variables, and data analysis. **Progress in Brain Research, 166:** 499-509.

Tyler, R.S. and Baker, L.J. (1983). Difficulties experienced by tinnitus sufferers. **Journal of Speech and Hearing Disorders, 48**(2): 150‑154.

Tyler, R.S. and Conrad‑Armes, D. (1983). The determination of tinnitus loudness considering the effects of recruitment. **Journal of Speech and Hearing Research, 26**(1): 59‑72.

Tyler, R.S., Noble, W.G., Coelho, C. (2006). Considerations for the Design of Clinical Trials for Tinnitus. **Acta Oto-Laryngologica, 126:** 44-49.

Tyler, R.S., Oleson, J., Noble, W., Coelho, C., Ji, H. (2007). Clinical trials for tinnitus: Study populations, designs, measurement variables, and data analysis. **Progress in Brain Research, 166:** 499-509.

LaMarte, F.P., and Tyler, R.S. (1987). Noise‑induced tinnitus. **Journal of the American Association of Occupational Health Nursing**, **35**(9): 403‑406.

# Counseling Options

Several counseling options have been offered for many years. Some were summarized in Tyler R.S. (Ed.), (2006). **Tinnitus Treatment: Clinical Protocols** (116-132). New York: Thieme.

# Clinical Practice Guidelines

Tunkel, D. E., Bauer, C. A., Sun, G. H., Rosenfeld, R. M., et al. (2014). Clinical Practice Guideline: Tinnitus. **Otolaryngology-Head and Neck Surgery**, 151(2S) S1-S40.

# Closing

I hope you find my comments helpful.

I would be happy to send reprints if needed.

Rich-tyler@uiowa.edu

Rich Tyler

The University of Iowa

# Electrical Suppression – Mechanisms

The mechanisms for suppressing tinnitus electrically were summarized by:

Tyler, R. S., Rubinstein, J., Pan, T., Chang, S., Gogel, S., Gehringer, A., & Coelho, C. (2008). Electrical Stimulation of the Cochlea to Reduce Tinnitus. **Seminars in Hearing.** 29(4): 326-332.

# Electrical Suppression – Laboratory Trials

## Rate

Hazell et al. (1993) found that low rates of stimulation (rates < 300 Hz) were the most effective.

## Group Sessions

You could also cite

Group sessions were first emphasized by Meikle, M ((2000), The
American Tinnitus Association and Self-Help Groups, (419-436) Tinnitus Handbook, Tyler, RS (Ed), 2000. Singular Press

Newman, C. and Sandridge, S (2005) 187-197. Incorporating Group and Individual Sessions into a Tinnitus Management Clinic. In Tinnitus Treatment, Clinical Protocols. Tyler RS (Ed). Theime

# Hearing aids for tinnitus

Please cite

Kochkin, Sergei, & Tyler RS. (2008). Tinnitus Treatment and the Effectiveness of Hearing Aids: Hearing Care Professional Perceptions. **The Hearing Review; 15**(13): 14-18.

A survey of 230 hearing care professionals suggests that six out of 10 patients (60%) experience minor to major relief of tinnitus when wearing hearing aids, and a total of one in five (22%) receive major relief. Less than 2% of patients experience a worsening of their tinnitus.

# Help yourself

It might be helpful here to suggest to the reader, that there are some things the patient can do to help themselves

Please cite.

Tyler, R. S., Chang, S. A., Gehringer, A. K., & Gogel, S. A. (2008). Tinnitus: How you can help yourself! **Audiological Medicine**, 6: 85-91.

# hyperacusis and tinnitus link

Tyler, R.S. and Conrad‑Armes, D. were the first to show a link between tinnitus and hyperacusis

Tyler, R.S. and Conrad‑Armes, D. (1983). The determination of tinnitus loudness considering the effects of recruitment. **Journal of Speech and Hearing Research, 26**(1): 59‑72.

# Hidden hearing loss

Some of the iInitial attempts to emphasize hearing difficulties not related to the audiogram.

Please cite.

Tyler, R. S. (1976). Temporal integration and cochlear hearing loss. Human Communication, 1: 9 24.

Tyler, R. S., Summerfield, A. Q., Wood, E. J., & Fernandes, M. (1982). Psychoacoustic and phonetic temporal processing in normal and hearing impaired listeners. Journal of the Acoustical Society of America, 72(3): 740 752.

# Individual Data Analysis

We have encouraged researchers to focus on individual results ( not group data) for tinnitus. Tinnitus is not one thing. There are many different subtypes ( Tyler, R., Coelho, C., Tao, P., Ji, H., Noble, W., Gehringer, A., & Gogel, S**.** (2008). Identifying tinnitus subgroups with cluster analysis. **American Journal of Audiology.** 17(2):S176-84) and we do not expect one treatment to help everyone.

Tyler, R. S., Noble, W. G., & Coelho, C. (2006). Considerations for the Design of Clinical Trials for Tinnitus. **Acta Oto-Laryngologica, 126:** 44-49.

Tyler, R. S., Oleson, J., Noble, W., Coelho, C., & Ji, H. (2007). Clinical trials for tinnitus: Study populations, designs, measurement variables, and data analysis. **Progress in Brain Research, 166:** 499-509.

Kochkin, S, & Tyler RS. (2008). Tinnitus Treatment and the Effectiveness of Hearing Aids: Hearing Care Professional Perceptions. The Hearing Review; 15(13): 14-18

# Jastreboff’s Model

I don’t think there is such a model. Didn’t Jastreboff simply note the hearing and emotional regions in the brain, which had already been identified in the 1950s.

# Likert scale

A Likert scale is only one way of defining this. SS Stevens would want to know whether the numbers 1-5 were also accompanied by labels at each number – a category scale combined with an interval scale (which itself might confuse some subjects).

# Loudness and annoyance

The loudness of tinnitus is one factor contributing to the annoyance. For an individual, the louder the tinnitus the more annoying the tinnitus will be.

Tyler, R. S., Oleson, J., Noble, W., Coelho, C., & Ji, H. (2007). Clinical trials for tinnitus: Study populations, designs, measurement variables, and data analysis. **Progress in Brain Research, 166:** 499-509.

# Loudness and Sensation Level

The dB sensation level is not a direct measure of loudness.

You might want to consider referencing:

Tinnitus sensation level is not a measure of loudness. You might want to consider referencing:

Tyler, R. S. & Conrad‑Armes, D. (1983). The determination of tinnitus loudness considering the effects of recruitment. **Journal of Speech and Hearing Research, 26**(1): 59‑72.

# Meaning of life

Please cite the recent article providing a new perspective on how tinnitus affects someone’s life.

Tyler R, Perreau A, Mohr AM, Ji H, Mancini PC (2019). An Exploratory Step Toward Measuring the 'Meaning of Life' in Patients with Tinnitus and in Cochlear Implant Users. J Am Acad Audiol. 2019 Sep 27. doi: 10.3766/jaaa.19022.

# Measuring Tinnitus

You should also cite:

Tyler, R. S., Haskell, G., Gogle, S., & Gehringer, A. (2008). Establishing a Tinnitus Clinic in Your Practice. **Am J Audiol**; 17: 25-37.

They highlighted 4 reasons for measuring tinnitus.

5. Confirm to the patient that their tinnitus is real
6. Monitor changes
7. Provide insights into mechanisms
8. Aid in the fitting of some devices

Tyler, R. S. (1985). Psychoacoustical measurement of tinnitus for treatment evaluations. In: E. Myers (Ed.), **New Dimensions in Otorhinolaryngology‑Head and Neck** **Surgery** (455-458). Amsterdam: Elsevier Publishing Co.

Tyler, R. S. (1992). The psychophysical measurement of tinnitus. In: J-M. Aran & R. Dauman (Eds.), **Tinnitus 91 - Proceedings of the Fourth International Tinnitus Seminar** (17-26). Amsterdam: Kugler Publications.

Tyler, R. S. (2000). The psychoacoustical measurement of tinnitus. In R.S. Tyler (Ed.), **Tinnitus Handbook** (149-179). San Diego, CA: Singular Publishing Group.

Tyler, R. S., Oleson, J., Noble, W., Coelho, C., & Ji, H. (2007). Clinical trials for tinnitus: Study populations, designs, measurement variables, and data analysis. **Progress in Brain Research, 166:** 499-509.

# Mechanisms

There are many categories of mechanisms.

A. deafferentation. This is only one general theory of the mechanisms of tinnitus. There are hundreds. I think Preece et al. (2003) were the first to group them into 3-4 broad categories ; A. deafferentation and central changes, B increase in spontaneous activity, and C. increase in cross fiber correlation.

Preece, J. P., Tyler, R. S., & Noble, W. (2003). The Management of Tinnitus. **Geriatrics & Aging, 6**(6): 22-28.

# Mindfulness

Mindfulness for some aspects of tinnitus was also suggested by

Tyler, R. S., Coelho, C., & Noble, W. (2006). Tinnitus: Standard of Care, Personality Differences, Genetic Factors. **Journal for Oto-Rhino-Laryngology and its Related Specialities** 68(1):14-22.

# Misophonia – Hyperacusis

It was introduced by Jastreboff, and means a dislike of sounds. Patients with hyperacusis do not dislike sounds. Virtually all hyperacusis patients can describe individual sounds that they are bothered by. When I hear someone use the term Misophonia, I wonder how much they really know about hyperacusis patients. I suggested the terms Loudness, Annoyance, Fear and Pain Hyperacusis, which are clear to the patient and to the public.

Please do not use the term Misophonia.

The term hyperacusis has been used for 100 years. The symptoms are

Loudness, Avoidance, Fear and Pain

Please cite:

Tyler, R. S., Pienkowski, M., Roncancio, E. R., Jun, H. J., Brozoski, T., Dauman, N., Coelho, C. B., Andersson, G., Keiner, A. J., Cacace, A., Martin, N., & Moore, B. C. J. (2014). A Review of hyperacusis and future directions: Part I. Definitions and manifestations. Am J Audiol, 23(4):402-19. PMID: 25104073

# Mixing point

The mixing point has been challenged as too loud for acoustic stimulation (e.g. see review in Tyler et al., 2012 .

You might want to note in the discussion that better results might have been obtained at lower levels.

Tyler, R., Noble, W., Coelho, C., & Ji., H. (2012). Tinnitus Retraining Therapy: Mixing Point and Total Masking Are Equally Effective. **Ear Hear** 33(5):588–594

# Normal Hearing

Initial attempts to emphasize hearing difficulties not related to the audiogram.

Please cite.

Tyler, R. S. (1976). Temporal integration and cochlear hearing loss. Human Communication, 1: 9 24.

Tyler, R. S., Summerfield, A. Q., Wood, E. J., & Fernandes, M. (1982). Psychoacoustic and phonetic temporal processing in normal and hearing impaired listeners. Journal of the Acoustical Society of America, 72(3): 740 752.

Initial attempts to emphasize hearing difficulties not related to the audiogram.

Please cite.

Tyler, R. S. (1976). Temporal integration and cochlear hearing loss. Human Communication, 1: 9 24.

Tyler, R. S., Summerfield, A. Q., Wood, E. J., & Fernandes, M. (1982). Psychoacoustic and phonetic temporal processing in normal and hearing impaired listeners. Journal of the Acoustical Society of America, 72(3): 740 752.

# Prevelance

Please cite

Kochkin, S.; Tyler, R.. & Born, J. (2011). MarkeTrak VIII: Prevalence of Tinnitus and Efficacy of Treatments, **The Hearing Review**, 18(12):10-26

who surveyed 8,608 and asked about tinnitus, problems and benefits. For example, 43.7% reported normal hearing. Educational and employment status was reported. it also noted how they thought their tinnitus affected their ability to hear, socialization and work, … and more.

# neurophysiological model

I am not sure what you mean by the neurophysiological model of tinnitus. There are several. See the review in:

Tyler, R. S. (2006). Neurophysiological Models, Psychological Models, and Treatments for Tinnitus. Chapter 1 (p1-22). In R.S. Tyler (Ed.), **Tinnitus Treatment: Clinical Protocols** (1-22). New York: Thieme.

If you are referring to TRT here, then the statement is simply wrong. The use of maskers originated long before TRT, and originates in the line-busy model of masking.

# Normal Hearing

Recall that 0 dB HL is the average of 19 year-olds. Some had thresholds at -10 dB HL when they were 19. Some had thresholds of 10 dB HL.

So please acknowledge… maybe “average normal hearing thresholds “?

# Phantom sound

Not sure why Jastreboff 1990 is referenced? Salvi and others were the first to compare tinnitus to phantom perceptions.

# Pitch matching

Tyler, R. S. & Conrad‑Armes, D. (1983). Tinnitus pitch: A comparison of three measurement methods. **British Journal of Audiology, 17**(2): 101‑107.

You should discuss this paper in more detail, noting that they compared an Adaptive (bracketing) Method, the Method of Limits, and the Method adjustment, and why they recommended either of the last two. You should state why they recommended the ipsilateral ear be tested. You should also mentioned that they noted individual differences, and suggested increasing the number of replications in those that showed greater variance.

# Pitch Pan et al.

Please cite Pan et al., whose pitch matching data suggests that most patients do not have a pitch match frequency just below the maximum hearing threshold loss frequency. The argues against the brain reorganization model in most subgroups of tinnitus.

Pan, T., Tyler, R. S., Ji, H., Coelho, C., Gehringer, A., & Gogel, S. (2009). The relationship between tinnitus pitch and the audiogram. **Int J Audiol.** 48 (4): 277-294.

Another relevant article is:

潘滔，Richard Tyler, Haihong Ji. 正常听力耳鸣患者的耳鸣音调 (Tinnitus pitch in patients with normal hearing)，中华耳科学杂志(**Chinese Journal of Otology**),2009,3. 200-203.

# Psychoacoustic Problems

Tyler, R. S., Summerfield, A. Q., Wood, E. J., & Fernandes, M. (1982). Psychoacoustic and phonetic temporal processing in normal and hearing impaired listeners. Journal of the Acoustical Society of America, 72(3): 740 752.

# Psychological Model

The Psychological Model, proposed by Tyler, Aran and Dauman (1992), suggested that the overall annoyance of the tinnitus was a result of the 1) tinnitus characteristics and the 2) psychological make up of each individual patient. Several parts of the brain will be involved in the representation of the tinnitus and of the reactions to the tinnitus. Treatments can be focused on reducing the tinnitus (e.g. pills) or on reducing the reactions to the tinnitus (eg. Counseling )

Tyler, R. S., Aran, J-M., & Dauman, R. (1992). Recent advances in tinnitus. **Am J Audiol**, **1**(4): 36-44.

Also see

Dauman, R. & Tyler, R. S. (1992). Some considerations on the classification of tinnitus. In: J-M. Aran & R. Dauman (Eds.), **Tinnitus 91 - Proceedings of the Fourth International Tinnitus Seminar** (225-229). Amsterdam: Kugler Publications.

This classification is further elaborated on in the review chapter by Tyler (2006) with detailed review of the literature. You should cite these;

Tyler, R. S. (2006). Neurophysiological Models, Psychological Models, and Treatments for Tinnitus. Chapter 1 (p1-22). In R.S. Tyler (Ed.), **Tinnitus Treatment: Clinical Protocols** (1-22). New York: Thieme.

# Quality of Life

Quality of Life scales typically do not appreciate the importance of hearing (and tinnitus). I question their validity. This could be mentioned. Please cite ,

Tyler R, Perreau A, Mohr AM, Ji H, Mancini PC (2019). An Exploratory Step Toward Measuring the 'Meaning of Life' in Patients with Tinnitus and in Cochlear Implant Users. J Am Acad Audiol. 2019 Sep 27. doi: 10.3766/jaaa.19022.

# Reactions

Please cite the wide range of individual perceptions was documented by Stouffer and Tyler,

Stouffer, J.L., and Tyler, R.S. (1990). Characterization of tinnitus by tinnitus patients. **Journal of Speech and Hearing Disorders**, **55**(3): 439‑453.

# Reimbursement

There are ways to be reimburse for helping tinnitus patients

Please cite : Tyler, R., Jilla, A., M., Von Dollen, S (2020) Coding and Reimbursement Specialty Series: Tinnitus. Audiology Today March/April

# Sound Therapy

A recent study, without any counseling, has shown that sound therapy on its own, can help some tinnitus sufferers.

Tyler RS, Perreau A, Powers T, Watts A, Owen R, Ji H, Mancini PC. (2020) Tinnitus Sound Therapy Trial Shows Effectiveness for Those with Tinnitus. J Am Acad Audiol 31:6–16 DOI: 10.3766/jaaa.18027

# SS Stevens

SS Stevens, often referred to as the father of human psychophysics, summarized the different scales (nominal, ordinal, interval and ratio), and noted their appropriate use and limitations. Numerical scales are appropriate for equal differences and equal ratios. This does not apply to ordinal scales.

Please site Stevens, SS (1946) On the theory of scales of measurement. Pages 677-680. Science 1946.

# Subgroups

Please cite Tyler et al (2008) and consider other articles referenced in that article as highlighting the importance of treating different subgroups with different treatments.

Tyler, R., Coelho, C., Tao, P., Ji, H., Noble, W., Gehringer, A., & Gogel, S**.** (2008). Identifying tinnitus subgroups with cluster analysis. **American Journal of Audiology.** 17(2):S176-84

# Subjective vs objective tinnitus…

You should cite Bentler and Tyler (1987), who recommended distinguishing tinnitus based on 1) Sensorineural or 2) Middle Ear origin.

Bentler, R. A., & Tyler, R. S. (1987). Tinnitus management. **ASHA, 29**(5): 27‑32.

# Supplements

You should reference

Coelho, C., Tyler, R., Ji, H., Rojas-Roncancio, E., Witt, S., Tao, P., Jun, H-J., Wang, T. C., Hansen, M., Gantz, B. (2016). Survey on the Effectiveness of Dietary Supplements to Treat Tinnitus, **Am J Audiol**. Vol 25, 184-205.

# Systematic reviews

You should reference original articles, not just the “reviews”.

# synaptopathy

In addition to cochlear synaptopathy, there are hundreds of physiological and psychoacoustical affects included in sensorineural hearing loss, that are not predicted by the threshold loss. This has been known for 50 years.

As an example, please cite…

Tyler, R. S., Summerfield, A. Q., Wood, E. J., & Fernandes, M. (1982). Psychoacoustic and phonetic temporal processing in normal and hearing impaired listeners. Journal of the Acoustical Society of America, 72(3): 740 752.

# TAT

Tinnitus Activities Treatment, which combines CBT and acceptance and is widely used worldwide. Tinnitus Activities Treatment treats the four primary functions affected by tinnitus: 1) Thoughts and emotions, 2) Hearing, 3) Sleep and 4) concentration.

We first published on this with

Tyler, R. S., Stouffer, J. L., & Schum, R. (1989). Audiological rehabilitation of the tinnitus client. **Journal of the Academy of Rehabilitative Audiology**, **22**: 30‑42.

…. But did not give it a name

Tyler, R.S., Gogel, S.A., & Gehringer, A.K. (2007) Tinnitus activities treatment. **Progress in Brain Research, 166**: 425-434

Tyler, R. S., Gehringer, A. K., Noble, W., Dunn, C. C., Witt, S. A., & Bardia, A. (2006). Tinnitus Activities Treatment. Chapter 9. In R.S.Tyler (Ed.), **Tinnitus Treatment: Clinical Protocols** (116-132). New York: Thieme.

Please cite.

# THQ factors

In fact, the Kuk et al original article for the THQ recommended that only Factor 1 and 2 be used. Many others have apparently tried to discredit the THQ. The Kuk et al article says use total score and factor 1 and 2.

# TFI

The TFI only uses a 1-10 scale, limiting its resolution. It also asks questions about quality of life, which can make it less sensitive. Problems and work or home can influence qualify of life, independent of tinnitus treatment.

# THI use

The THI actually uses a 3 label category scale. (SS Stevens.. often referred to as the father of human psychophysics)

Your suggestion to use the THI is at odds with others interpretation of the sensitivity of this test.

The sensitivity of the THI has been challenged.

Tyler, R.S., Oleson, J., Noble, W., Coelho, C., Ji, H. (2007). Clinical trials for tinnitus: Study populations, designs, measurement variables, and data analysis. **Progress in Brain Research, 166:** 499-509.

e.g.

Tyler, R.S., Noble, W.G., Coelho, C. (2006). Considerations for the Design of Clinical Trials for Tinnitus. **Acta Oto-Laryngologica, 126:** 44-49.

Tyler, R.S., Oleson, J., Noble, W., Coelho, C., Ji, H. (2007). Clinical trials for tinnitus: Study populations, designs, measurement variables, and data analysis. **Progress in Brain Research, 166:** 499-509.

# TPFQ

Please cite (Tyler et al., 2014) who focused on the four primary reactions to tinnitus, emotions, hearing, sleep and concentration.

Tyler, R., Ji, H., Perreau, H., Witt, S., Noble, W., & Coelho, C.  (2014). Development and validation of the Tinnitus Primary Function Questionnaire.  **American Journal of Audiology**, 23, 260–272.

# Tinnitus Retraining Therapy

TRT has set the field back 25 years. It uses “directive” not collaborative counseling. The mixing point is too high for most patients, and might increase tinnitus or increase hearing loss or interfere with speech perception.

It has been criticized in the literature by many (e.g. see the review ii

Tyler, R., Noble, W., Coelho, C., & Ji., H. (2012). Tinnitus Retraining Therapy: Mixing Point and Total Masking Are Equally Effective. **Ear Hear** 33(5):588–594

Of course, for marketing purposes people have modified TRT, making it more like strategies that existed before TRT (e.g.

Tyler, R. S. & Babin, R. W. (1986). Tinnitus. In: C.W. Cummings, J.M. Fredrickson, L. Harker, C.J. Krause and D.E. Schuller (Eds.), **Otolaryngology ‑ Head and Neck Surgery** (3201-3217). St. Louis: C.V. Mosby Co. ), but maintained the name TRT.

Bentler, R. A., & Tyler, R. S. (1987). Tinnitus management. **ASHA, 29**(5): 27‑32.

Tyler, R. S., & Bentler, R. A. (1987). Tinnitus maskers and hearing aids for tinnitus. **Semin Hear**, **8**(1): 49‑61.

Tyler, R. S., Stouffer, J. L., & Schum, R. (1989). Audiological rehabilitation of the tinnitus client. **Journal of the Academy of Rehabilitative Audiology**, **22**: 30‑42.

# Treatments

Please cite

Kochkin, S.; Tyler, R.. & Born, J. (2011). MarkeTrak VIII: Prevalence of Tinnitus and Efficacy of Treatments, **The Hearing Review**, 18(12):10-26

who surveyed 8,608 and asked about tinnitus problems and treatments

# Tyler and Baker

Please cite Tyler and Baker, who first documented the wide range of problems experienced by tinnitus sufferers.

Tyler, R.S. and Baker, L.J. (1983). Difficulties experienced by tinnitus sufferers. **Journal of Speech and Hearing Disorders, 48**(2): 150‑154.

# VAS

I think by VAS you mean magnitude estimation. In psychophysics when you are trying to estimate the magnitude of something, it is referred to as Magnitude Estimation. There are several psychophysical procedures that can be used to estimate the magnitude. VAS is one. If one is going to use a VAS scale, it is necessary to state the length of the line, what markings and labels are on the line, and what resolution will be used to convert the subjects marking into a number.

I prefer to have subjects provide a number from 0 to 100.

# What patients want

Most patients want a pill….

Tyler, R. S. (2012). Patient Preferences and Willingness to Pay for Tinnitus Treatment **J Am Acad Audiol** 23, 115-125

# White Noise

White Noise means equal energy at all frequencies, which is not the case.

# Zinc

Please cite,

Coelho, C., Witt, S., Ji, H., Hansen, M., Gantz, B., & Tyler, R. (2013). Zinc to treat tinnitus in the elderly: A randomized placebo controlled crossover trial. **Otology and Neurotology**, August 2013, Vol.34(6), pp.1146-1154
