# Supplementary material for: The effect of caffeine on tinnitus: Randomized triple-blind placebo-controlled clinical trial
Source: PLoS One. 2021 Sep 20;16(9):e0256275. doi: 10.1371/journal.pone.0256275 (PMC8452027; doi:10.1371/journal.pone.0256275)
Supplement: Attachment — Submitted filename: Response to Reviewers.docx [file pone.0256275.s007.docx]

- Response to Reviewers

More like once again to thank the excellent contributions made about our study. All of them have been taken into account. An additional statistic was performed in order to meet the requests and special attention was given to the writing of the manuscript. Below are the answers to each consideration made.

**Reviewer 1:**

- The comparison was conducted among groups (difference of the differences) through the non-parametric Mann-Whitney test. This analysis has been added in the "Results", pages 13-16, lines 252-255, 2-271-275, 295-299; and in Appendix 2.
- THI was best described in terms of composition and analysis in "Methods" ("Questionnaires and exams"), page 7, lines 147-154. This questionnaire was subdivided into three scales - Emotional, Functional and Catastrophic - (Newman et al., 1996), however, a study carried out seeking to analyze the factor structure demonstrated that the THI total score can serve as a robust measure of tinnitus distress and recommended using only the total score both in research and in clinical practice (Baguley; Andreson, 2003). Thus, the authors decided to perform the analysis of the total score without presenting the values by sub-scale. The classification of degrees of tinnitus has been added (Newman et al., 1996): “Results”, Table 1, page 11. As suggested by one of the reviewers, the analysis of the items corresponding to the "lack of control" (questions 8 and 19 of the THI) was added since this represents the areas which may produce the most dramatic effects if changes are observed: “Results”, page 13, lines 244-248
- The POMS questionnaire was described for composition and analysis in “Methods" ("Questionnaires and exams"), page 8, lines 156-162. The meaning for the abbreviation "TMD" has been added.
- Added a clear description of the study hypothesis: "Introduction", page 04, lines 71-72
- The eligibility criteria were best described: “Methods”, page 5, lines 85-86
- Specification was provided that the value in parentheses referred to the p-value: "Results", page 13, lines 234-239
- Added the description that the values in phase 3 were lower than in phase 1: "Results", page 13, line 239
- The IQR for THI and VAS has been added: "Results”, page 13, line 243 and page 14, lines 250-251
- The test legend used in Table 2 was adjusted: chapter "Results", page 13/14, line 229
- An analysis of the correlation between caffeine consumption and the variables analyzed in the study was added: pages 13-16, lines 252-255, 2-271-275, 295-299; and in Appendix 2.
- Article writing was revised trying to let it more cohesive and clear

**Reviewer 2:**

- The description of the placebo was added in the abstract, page 2, line 25
- The choice of a restricted caffeine diet for 24 hours before the study was based on the pharmacology of the substance (presented in the chapter "Methods", page 6, lines 109-114 and in previous studies that sought to investigate the action of caffeine (Hughes, 1991)
- A more detailed explanation of the Visual Analog scale (VAS) has been added: "Methods" ("Questionnaires and exams"), page 8, lines 153-154.
- Added the explanation of what is the acufenometry: Abstract, page 2, lines 29-30
- The description of what was considered to be a clinically relevant hearing threshold change was obtained in the chapter "Discussion", page 19, lines 376-379
- Added the Psychological Model of Tinnitus: "Introduction", page 3, lines 45-46
- THI was best described in terms of composition and analysis in "Methods" ("Questionnaires and exams"), page 7, lines 147-154. This questionnaire was subdivided into three scales - Emotional, Functional and Catastrophic - (Newman et al., 1996), however, a study carried out seeking to analyze the factor structure demonstrated that the THI total score can serve as a robust measure of tinnitus distress and recommended using only the total score both in research and in clinical practice (Baguley; Andreson, 2003). Thus, the authors decided to perform the analysis of the total score without presenting the values by sub-scale. The classification of degrees of tinnitus has been added (Newman et al., 1996): “Results”, Table 1, page 11. As suggested by one of the reviewers, the analysis of the items corresponding to the "lack of control" (questions 8 and 19 of the THI) was added since this represents the areas which may produce the most dramatic effects if changes are observed: “Results”, page 13, lines 244-248
- The importance of performing psychoacoustic measurements of tinnitus was presented in the chapter "Introduction", page 3, lines 61-62. The loudness measurement was performed as described by Henry and Zaugg (2005), although the authors recognize the limitations arising from the measures in dB sensation level (Tyler and Conrad, 1983), and this dimension discussed in the "Discussion" , page 19, lines 363-367
- The average duration of the study was added, and the duration in each phase varied according to the individual's response: “Methods”, page 7, line 133
- Added food description: mate herb: “Methods”, page 7, line 144
- The description of the protocols used was revised. Methods Chapter (Questionnaires and exams), pages 7-9
- The 6-month recruitment period was best described: “Results”, page 10, line 214
- The description of normal hearing has been added: “Methods”, page 8, lines 169-170
- The error (3000Hz) has been fixed: Chapter Discussion, page 18, line 355
