# Supplementary material for: The effect of caffeine on tinnitus: Randomized triple-blind placebo-controlled clinical trial
Source: PLoS One. 2021 Sep 20;16(9):e0256275. doi: 10.1371/journal.pone.0256275 (PMC8452027; doi:10.1371/journal.pone.0256275)
Supplement: Attachment — Submitted filename: Response to Reviewers.docx [file pone.0256275.s008.docx]

Response to Reviewers

The last suggested review on the 5th of July contains the same requests as the previous review of the 10th of March 2021.The requests were met in the submitted manuscript and indicated on the document "Response to reviewers" as requested.

**• Line 21 -Define what the placebo is in the abstract.**

The description of the placebo was added in the abstract, page 2, line 25

**• Line 26 - 24 hours does not seem long as control…? Explain what this is.**

The choice of a restricted caffeine diet for 24 hours before the study was based on the pharmacology of the substance (presented in the chapter "Methods", page 6, lines 109-114 and in previous studies that sought to investigate the action of caffeine (Hughes, 1991)

• **Line 27 - VAS here and elsewhere. I think by VAS you mean magnitude estimation. In psychophysics when you are trying to estimate the magnitude of something, it is referred to as Magnitude Estimation. There are several psychophysical procedures that can be used to estimate the magnitude. VAS is one. If one is going to use a VAS scale, it is necessary to state the length of the line, what markings and labels are on the line, and what resolution will be used to convert the subjects marking into a number. I prefer to have subjects provide a number from 0 to 100.**

A more detailed explanation of the Visual Analog scale (VAS) has been added: "Methods" ("Questionnaires and exams"), page 8, lines 153-154.

• **Line 29 - Most readers will not know what acufenometry is.**

Added the explanation of what is the acufenometry: Abstract, page 2, lines 29-30

**• Line 35 - How do you know what is clinically relevant?**

The description of what was considered to be a clinically relevant hearing threshold change was obtained in the chapter "Discussion", page 19, lines 376-379

• **Line 48 - Please cite the Psychological Model, proposed by Tyler, Aran and Dauman(1992), suggested that the overall annoyance of the tinnitus was a resultof the 1) tinnitus characteristics and the 2) psychological make up of eachindividual patient. Several parts of the brain will be involved in therepresentation of the tinnitus and of the reactions to the tinnitus. Treatments can be focused on reducing the tinnitus (e.g. pills) or onreducing the reactions to the tinnitus (eg. Response to Reviewers Counseling )Tyler, R. S., Aran, J-M., & Dauman, R. (1992). Recent advances intinnitus. Am J Audiol, 1(4): 36-44.**

Added the Psychological Model of Tinnitus: "Introduction", page 3, lines 45-46

**• Line 52 - The THI actually uses a 3 label category scale. (SS Stevens.. often referred to as the father of human psychophysics) Please discuss in the results, the sensitivity of the THI has been challenged. Tyler, R.S., Oleson, J., Noble, W., Coelho, C., Ji, H. (2007). Clinical trials for tinnitus: Study populations, designs, measurement variables, and data analysis. Progress in Brain Research, 166: 499- 509.**

THI was best described in terms of composition and analysis in "Methods" ("Questionnaires and exams"), page 7, lines 147-154. This questionnaire was subdivided into three scales - Emotional, Functional and Catastrophic - (Newman et al., 1996), however, a study carried out seeking to analyze the factor structure demonstrated that the THI total score can serve as a robust measure of tinnitus distress and recommended using only the total score both in research and in clinical practice (Baguley; Andreson, 2003). Thus, the authors decided to perform the analysis of the total score without presenting the values by sub-scale. The classification of degrees of tinnitus has been added (Newman et al., 1996): “Results”, Table 1, page 11. As suggested by one of the reviewers, the analysis of the items corresponding to the "lack of control" (questions 8 and 19 of the THI) was added since this represents the areas which may produce the most dramatic effects if changes are observed: “Results”, page 13, lines 244- 248; “Discussion”, page 17, lines 331-339

**• Line 60 - Measuring tinnitus. The dB sensation level is not a direct measure of loudness. Please reference Tyler, R. S. & Conrad Armes, D. (1983). The determination of tinnitus loudness considering the effects of recruitment. Journal of Speech andHearing Research, 26(1): 59 72. You should also cite: Tyler, R. S., Haskell, G., Gogle, S., & Gehringer, A. (2008). Establishing aTinnitus Clinic in Your Practice. Am J Audiol; 17: 25-37. They highlighted 4 reasons for measuring tinnitus. 1. Confirm to the patient that their tinnitus is real 2. Monitor changes 3. Provide insights into mechanisms 4. Aid in the fitting of some devices Tyler, R. S. (1985). Psychoacoustical measurement of tinnitus fortreatment evaluations. In: E. Myers (Ed.), New Dimensions inOtorhinolaryngology Head and Neck Surgery (455-458). Amsterdam:Elsevier Publishing Co. Tyler, R. S. (1992). The psychophysical measurement of tinnitus. In: J-M. Aran & R. Dauman (Eds.), Tinnitus 91 - Proceedings of the FourthInternational Tinnitus Seminar (17-26). Amsterdam: Kugler Publications. Tyler, R. S. (2000). The psychoacoustical measurement of tinnitus. InR.S. Tyler (Ed.), Tinnitus Handbook (149-179). San Diego, CA: SingularPublishing Group. Tyler, R. S., Oleson, J., Noble, W., Coelho, C., & Ji, H. (2007). Clinical trialsfor tinnitus: Study populations, designs, measurement variables, and dataanalysis. Progress in Brain Research, 166: 499- 509.**

The importance of performing psychoacoustic measurements of tinnitus was presented in the chapter "Introduction", page 3, lines 61-62. The loudness measurement was performed as described by Henry and Zaugg (2005), although the authors recognize the limitations arising from the measures in dB sensation level (Tyler and Conrad, 1983), and this dimension discussed in the "Discussion", page 19, lines 363-367; 369-372

• **Line 132 - Define the duration of the phases.**

The average duration of the study was added, and the duration in each phase varied according to the individual's response: “Methods”, page 7, line 133

• **Line 140 - Mate ??**

Added food description: mate herb: “Introduction”, page 4, line 66; “Methods”, page 7, line 144

• **Line 154 - Define the protocols so that some can replicate your study, a fundamental of science.**

The description of the protocols used was revised. Methods Chapter (Questionnaires and exams), pages 7-9

• **Line 197 - Not clear what the 6 month period stands for.**

The 6-month recruitment period was best described: “Results”, page 10, line 214-215
